# Single-cell RNA sequencing reveals markers of disease progression in primary cutaneous T-cell lymphoma

**DOI:** 10.1186/s12943-021-01419-2

**Published:** 2021-09-28

**Authors:** Katharina Rindler, Constanze Jonak, Natalia Alkon, Felix M. Thaler, Harald Kurz, Lisa E. Shaw, Georg Stingl, Wolfgang Weninger, Florian Halbritter, Wolfgang M. Bauer, Matthias Farlik, Patrick M. Brunner

**Affiliations:** 1 Department of Dermatology, Medical University of Vienna, Währinger Gürtel 18-20, 1090 Vienna, Austria; 2 St. Anna Children’s Cancer Research Institute (CCRI), Zimmermannplatz 10, 1090 Vienna, Austria

**Keywords:** Cutaneous lymphoma, Mycosis fungoides, Single-cell RNA sequencing, Gamma-delta lymphoma, Advanced-stage MF, Early-stage MF, Nonlesional MF

## Abstract

**Background:**

In early-stage mycosis fungoides (MF), the most common primary cutaneous T-cell lymphoma, limited skin involvement with patches and plaques is associated with a favorable prognosis. Nevertheless, approximately 20–30% of cases progress to tumors or erythroderma, resulting in poor outcome. At present, factors contributing to this switch from indolent to aggressive disease are only insufficiently understood.

**Methods:**

In patients with advanced-stage MF, we compared patches with longstanding history to newly developed plaques and tumors by using single-cell RNA sequencing, and compared results with early-stage MF as well as nonlesional MF and healthy control skin.

**Results:**

Despite considerable inter-individual variability, lesion progression was uniformly associated with downregulation of the tissue residency markers *CXCR4* and *CD69*, the heat shock protein *HSPA1A*, the tumor suppressors and immunoregulatory mediators *ZFP36* and *TXNIP*, and the interleukin 7 receptor (*IL7R)* within the malignant clone, but not in benign T cells. This phenomenon was not only found in conventional TCR-αβ MF, but also in a case of TCR-γδ MF, suggesting a common mechanism across MF subtypes. Conversely, malignant cells in clinically unaffected skin from MF patients showed upregulation of these markers.

**Conclusions:**

Our data reveal a specific panel of biomarkers that might be used for monitoring MF disease progression. Altered expression of these genes may underlie the switch in clinical phenotype observed in advanced-stage MF.

**Supplementary Information:**

The online version contains supplementary material available at 10.1186/s12943-021-01419-2.

## Introduction

Primary cutaneous T-cell lymphomas (CTCL) comprise a clinically and biologically heterogeneous group of malignancies arising from the clonal proliferation of skin-homing or skin-resident T cells [1]. The most frequent clinical entity is mycosis fungoides (MF), accounting for approximately 60% of all cases [2, 3]. In early disease stage, MF presents with patches and/or plaques, which often remain stable over many years without phenotypic changes or metastatic spread [4]. However, in approximately 20–30% of cases, MF progresses to advanced-stage disease by developing skin tumors and/or erythroderma, and ultimately disseminates to blood, lymph nodes and internal organs. This results in an unfavorable 5-year overall survival of less than 40% [5–8]. In the skin, the transition from early indolent to progressive disease is accompanied by a shift from a more type-1- towards a type-2-dominant immune signature of the tumor micromilieu [9], potential loss of certain T-cell antigens (CD2, CD3, CD5, CD7) [10] and/or loss of epidermotropism [11], but exact cell-intrinsic mechanisms and modes of intercellular communication driving this progression are still only insufficiently understood. Importantly, there are currently no molecular biomarkers available that can reliably predict disease outcome [12]. Thus, a better understanding of factors driving cancer cell progression is urgently needed. However, disease heterogeneity of MF, both on clinical and molecular levels, is a major challenge in this regard [12–15]. In this study, we profiled patches with longstanding history, and compared them with recently developed plaques or tumors within the same patient to overcome inter-individual variability. Follow-up skin biopsies were taken upon changes in phenotype or after therapeutic response. In addition, results were compared with early-stage disease, clinically unaffected (nonlesional) MF skin as well as control skin from healthy individuals. By using single-cell RNA sequencing (scRNA-seq) combined with T-cell receptor (TCR) sequencing, our data reveal a characteristic panel of six markers to be consistently downregulated in clonally expanded T cells of advancing MF lesions, as opposed to patches from indolent early-stage disease or clinically unaffected skin. Given the involvement of these markers in tissue retention, tumor suppression and inflammatory responses, they might be directly involved in mechanisms driving MF progression.

## Patients and methods

### Patient recruitment and sample processing

The study was conducted under a protocol approved by the Ethics Committee of the Medical University of Vienna, Austria (EK 1360/2018). Patients were recruited via our skin lymphoma clinic. Patient details are shown in Table 1. Punch biopsies were taken from clinically affected (lesional) and clinically unaffected (nonlesional) skin of MF patients, as well as healthy control individuals after obtaining written informed consent. For each described sample, one single 6 mm punch biopsy was taken and processed immediately. Each sample was cut into small pieces and digested in the C Tube (Miltenyi Biotec, Bergisch Gladbach, Germany) using the enzyme mix as provided by the Whole Skin Dissociation Kit for human (Miltenyi Biotec) in a total volume of 0,5 ml RPMI medium with enzymatic supplements as described in the manufacturer’s instructions. The sample was then incubated for a total of 1 hour in a water bath at 37 °C. The sample was then further dissociated using the gentleMACS Dissociator (Miltenyi Biotec) running the program h_skin_01. After termination of the program steps 10–14 were conducted as detailed in the manufacturer’s instructions. Finally, the cell pellet was resuspended in PBS with 0,08% BSA reaching a total volume of not more than 100 μl. A cell aliquot was stained with trypan blue and cell numbers and percentages of dead cells were assessed by manual counting. Samples with not more than 30% dead cells were then subjected to scRNA-seq processing as detailed below.

**Table 1:** Patient baseline characteristics at time of sampling

Subject ID	scRNA-seq	FACS/RT-PCR	Samples biopsied for scRNA-seq	Age	Sex	Ethnicity	Diagnosis	Large cell transformation	Disease duration (years)	Ongoing treatment	Previous treatments	Disease stage
MF309	x	x	Patch and tumor (trunk)	76	Male	Caucasian	CD4+ MF, CD30+	Present	Diagnosis 22 years ago; symptoms reported since age 37	ECP	Topical GCS, IFN alpha	IVA1 (T3N0M0B2)
MF309	x		Ulcerated tumor (follow-up lesion trunk) and nonlesional skin (trunk)	76			CD4+ MF, CD30+	Present		ECP	Brentuximab	IVA1 (T3N0M0B2)
MF311	x	x	Patch and plaque (trunk)	74	Female	Caucasian	CD4 + MF, CD30-	Absent	Diagnosis 2 years ago; symptoms reported since age 53	ECP	NB-UVB, bexarotene, PUVA, topical and systemic GCS, IFN alpha, alemtuzumab, local radiotheraphy (x ray)	IVA1 (T2N1M0B2)
MF311	x		Nonlesional skin (lower extremity)	74			CD4 + MF, CD30-	Absent		ECP		IVA1 (T2N1M0B2)
MF311	x		Erythroderma (follow-up lesion upper extremity)	75			CD4 + MF, CD30-	Absent		ECP, chlorambucil, systemic GCS		IVA1 (T4N1M0B2)
MF312	x		Patch and plaque (upper extremity)	55	Male	Caucasian	CD4 + MF, CD30+	Present	Diagnosis 6 years ago; symptoms since age 44	ECP, IFN alpha, acitretin	Topical GCS, NB-UVB, local radiotherapy (x ray)	IIB* (T2bN0M0B0)
MF312	x		Cleared (follow-up) lesion (upper extremity)	56				n.a.		ECP, chlormethine		IIB* (T1aN0M0B0)
MF318	x		Patch and plaque (thigh)	55	Female	Caucasian	gamma/delta MF, CD30+	Absent	Symptoms since age 52; recently diagnosed	none	Topical GCS and CNI, NB-UVB	IIB** (T3N0M0B0)
P65	x		Patch and non-lesional skin (upper extremity)	53	Male	Caucasian	CD4 + MF, CD30-	Absent	Symptoms for > 10 years; recently diagnosed	none	Topical GCS	IB (T2aN0M0B0)
P107	x		Patch lower back	39	Female	Caucasian	CD4−/CD8-MF, CD30+	Absent	Symptoms for > 20 years; diagnosed at age 37	none	Topical GCS	IA (T1bN0M0B0)
P107		x	Plaque lower back	39	Female	Caucasian	CD4−/CD8-MF, CD30+	Absent	Symptoms for > 20 years; diagnosed at age 37	none	Topical GCS	IA (T1bN0M0B0)
P138	x		Patch thigh	47	Male	Caucasian	CD4 + MF	Absent	Symptoms for > 20 years; recently diagnosed	none	Topical GCS	IA (T1aN0M0B0)
P73	x		Plaque and non-lesional skin (lower extremity)	82	Male	Caucasian	folliculotropic MF, CD30-	Absent	Diagnosis 6 years ago	none	PUVA, topical GCS, Re-PUVA, local radiotherapy	IIB (T3N0M0B0)
P84	x		Plaque (upper extremity)	75	Male	Caucasian	CD4 + MF, CD30-	Absent	Symptoms for 6 months; recently diagnosed	none	NB-UVB, topical GCS	IIB (T3N0M0B1)
P84	x		Cleared (follow-up) lesion (upper extremity)	75			CD4+ MF, CD30-	Absent		PUVA		IIB (T3N0M0B1)
P90	x		Patch and non-lesional skin (upper extremity)	75	Male	Caucasian	CD4 + MF	Absent	Diagnosis at age 64	none	topical GCS, PUVA, NB-UVB, bexarotene, acitretin	IB (T2aN0M0B0)
P15		x	Tumor trunk	59	Male	Caucasian	CD4 + MF, CD30+	Present	Diagnosis at age 56	Brentuximab vedotin	Topical and systemic GCS	IIB (T3N0M0B0)
P182		x	Patch and plaque (trunk)	49	Female	Caucasian	CD4 + CD8 + MF, CD30+	Absent	Onset of symptoms 4 months ago, recently diagnosed	none	none	IA (T1bN0M0B1)
P112	x		Healthy control skin	51	Female	Caucasian	n.a.	n.a.	n.a.	none	n.a.	n.a.
P115	x		Healthy control skin	48	Male	Caucasian	n.a.	n.a.	n.a.	none	n.a.	n.a.
P116	x		Healthy control skin	57	Female	Caucasian	n.a.	n.a.	n.a.	none	n.a.	n.a.
P121	x		Healthy control skin	44	Female	Caucasian	n.a.	n.a.	n.a.	none	n.a.	n.a.

*ECP* Extracorporeal photopheresis, *GCS* glucocorticosteroids, *IFN* interferon, *PUVA* psoralen ultraviolet A photochemotherapy, *NB-UVB* narrow band ultraviolet B phototherapy, *CNI* calcineurin inhibitor, *MF* mycosis fungoides; *history of skin tumors successfully treated with local radiotherapy, ** tumor was not biopsied, only patch and plaque

### Droplet-based single cell RNA sequencing

Single cell suspensions were subjected to scRNA-seq using the Chromium Single Cell Controller and Single Cell 5′ Library & Gel Bead Kit v1.1 (10X Genomics, Pleasanton, CA), according to the manufacturer’s protocol. A total of 25,000 cells were loaded for each sample on the Chip G (10X Genomics). cDNA was amplified using 13 PCR cycles. DNA concentration was assessed using the Qubit 1X dsDNA HS Assay Kit (Thermo Fisher Scientific) and fragment distribution was determined using the D5000 Screen Tape and the Tape Station 4150 system (Agilent Technologies, Santa Clara, CA). TCR αβ sequences were enriched from the cDNA using the respective reagents, and following the instructions of the VDJ Kit workflow by 10X Genomics with no adjustments. γδ TCR amplification from cDNA was performed by adhering to the primers and protocol procedure depicted in the ECCITE-seq workflow by Mimitou et al. [16]. Library preparation and enrichment was conducted following the manufacturer’s instructions (10X Genomics) and final quality control was carried out using the Qubit 1X dsDNA HS Assay Kit (Thermo Fisher Scientific) and the D1000 Screen Tape and the Tape Station 4150 system (Agilent Technologies). Sequencing was performed using the Illumina NovaSeq 6000 instrument and the SP platform in the 150 bp paired-end configuration.

For detailed methods on single-cell RNAseq data analyses, as well as immunofluorescence microscopy and quantitative RT-PCR procedures, please see supplemental methods.

## Results

### Single-cell RNA-seq mapping mycosis fungoides skin lesions

**Fig. 1:**
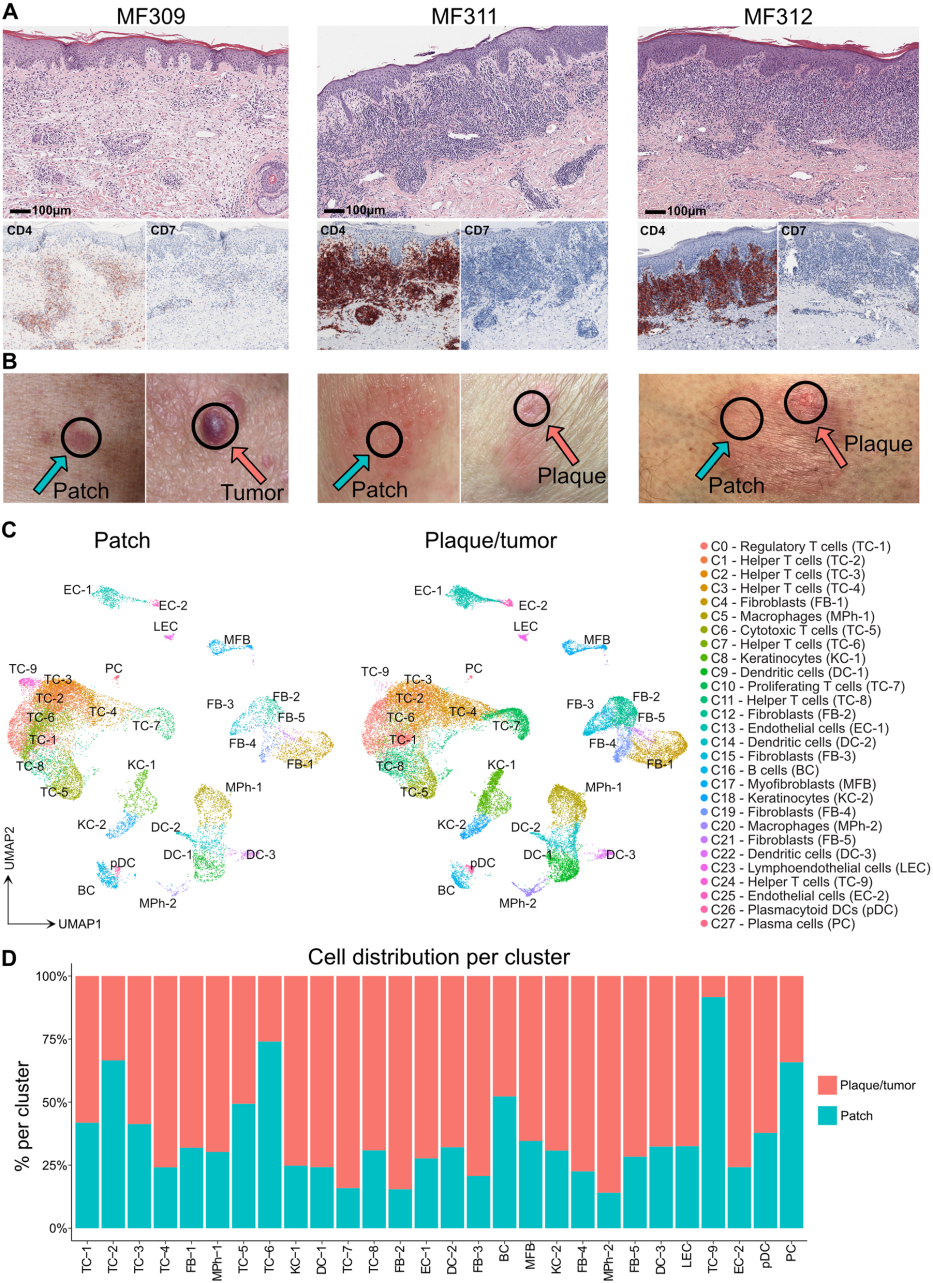
Comparison of patch with advancing plaque/tumor lesions in three individual MF patients. **A** Representative histopathological pictures of MF lesions from three different patients (biopsies for initial MF diagnosis). **B** Clinical pictures of MF lesions biopsied for single-cell RNA sequencing. Black circles indicate the biopsy location, turquoise and red arrows indicate patch and plaque/tumor lesions, respectively. **C** Overview of all cell clusters from 6 integrated MF patient samples (one patch and plaque/tumor lesion per patient). **D** Relative distribution of cells in patch vs. plaque/tumor lesions per cluster. TC T cells; BC B cells; KC keratinocytes; FB fibroblasts; DC dendritic cells; MPh macrophages; MFB myofibroblasts; EC endothelial cells; LEC lymphoendothelial cells; pDC plasmacytoid dendritic cells; PC plasma cells

We investigated three adult CTCL patients (MF309, MF311 and MF312, Table 1) suffering from advanced-stage MF (stage IIB or higher), previously confirmed by conventional histopathology (Fig. 1 A). Two patients (MF309, MF312) met histopathological criteria for large-cell transformation (Table 1), a feature associated with an unfavorable disease course [5]. At time of study inclusion, each patient showed both flat, stable patches of > 6 months duration, as well as recently developing palpable plaques and/or tumors. From each patient, we took biopsies from both a flat and a palpable lesion at the same time and from the same body region as adjacent as possible (Fig. 1 B), and performed single-cell RNA sequencing (scRNA-seq). Data were analyzed using the Seurat toolkit in R [17, 18]. After integration, we obtained a total of 47,172 cells, comprising 17,471 from three patches and 29,701 from three plaque/tumor lesions (Table S1). Data visualization using uniform manifold approximation and projection for dimension reduction (UMAP) [19] followed by unsupervised clustering depicted 28 distinct clusters (Fig. 1 C, S1A-B). We attributed cell identities using canonical markers and top upregulated genes (according to the smallest adjusted *p*-value and average log fold change) for each cluster compared to the rest of the dataset (Fig. S1C-D, Table S2). The largest cluster comprised *CD3D+* T cells, consisting of mutually exclusive populations of *CD8A+ GZMA+* cytotoxic (TC-5) and *CD4+* T-helper cells (TC-2: *CCR7*; TC-3: *CXCL13*; TC-4: *TNFRSF4*; TC-6: *CXCR4*; TC-9: *CCR7, MX1*), including *FOXP3+ CTLA4+* regulatory T cells (TC-1) and proliferating T cells (TC-7) (Fig. S1C, Table S2). By contrast, one *CD3D+* cluster (TC-8) seemed largely negative for both *CD4* and *CD8A* that was mainly derived from patient MF309 (Fig. S1C, Table S1). Re-examination of histopathological slides of this patient indeed suggested some MF cells to be negative for CD4 (data not shown). While type 1 *(IFNG)* and type 17/22 *(IL17A, IL22, IL26)* cytokines were mostly found in clusters TC-5/TC-8 and TC-8, respectively, the type-2 cytokine *IL13* was found at low frequencies across several clusters (Fig. S1E). A myeloid cell cluster contained dendritic cells (DC-1: *CD1A, CD1C*, and partly *CD207*; DC-2: *LYZ, IL1B*; DC-3: *LAMP3*) and macrophages (MPh1: *CD163, CCL18;* MPh2: *CPVL*). Smaller leukocyte clusters included B cells (*CD19*), plasmacytoid DCs (pDCs: *LILRA4*) and plasma cells (PC: *IGKC*). A larger population of *COL1A2+* fibroblasts contained five clusters (FB-1: *COL1A1, MFAP5, FBN1*-positive cells constituting the largest group [20]; FB-2: *CCL19, CXCL9;* FB-3: *POSTN, CCL2, COL6A5, COL18A1 and CCL19* cells previously described as inflammatory fibroblasts in AD [21]; FB-4: *APOD, CFD, APOE, CXCL12;* and FB-5: *COL1A1, SPARC*; Fig. 1 C, Fig. S1C-D, Table S2). We also found *KRT5+* keratinocytes (basal KC-1: *KRT15, KRT14;* suprabasal KC-2: *KRT1, KRT10*, Fig. S1D), myofibroblasts (MFB: *ACTA2*), blood endothelial cells EC-1 and EC-2 (*VWF*) and lymphoendothelial cells (LEC: *LYVE1, PDPN*) (Fig. S1C-D). These data demonstrate the presence of all major skin cell types and clusters in each individual sample, except for TC-9, which was found primarily in MF311 patch lesions (Fig. 1 D, Table S1, Fig. S2A-F).

### T-cell receptor (TCR) sequencing detects considerable inter-individual transcriptomic heterogeneity in the dominant T cell clone

**Fig. 2:**
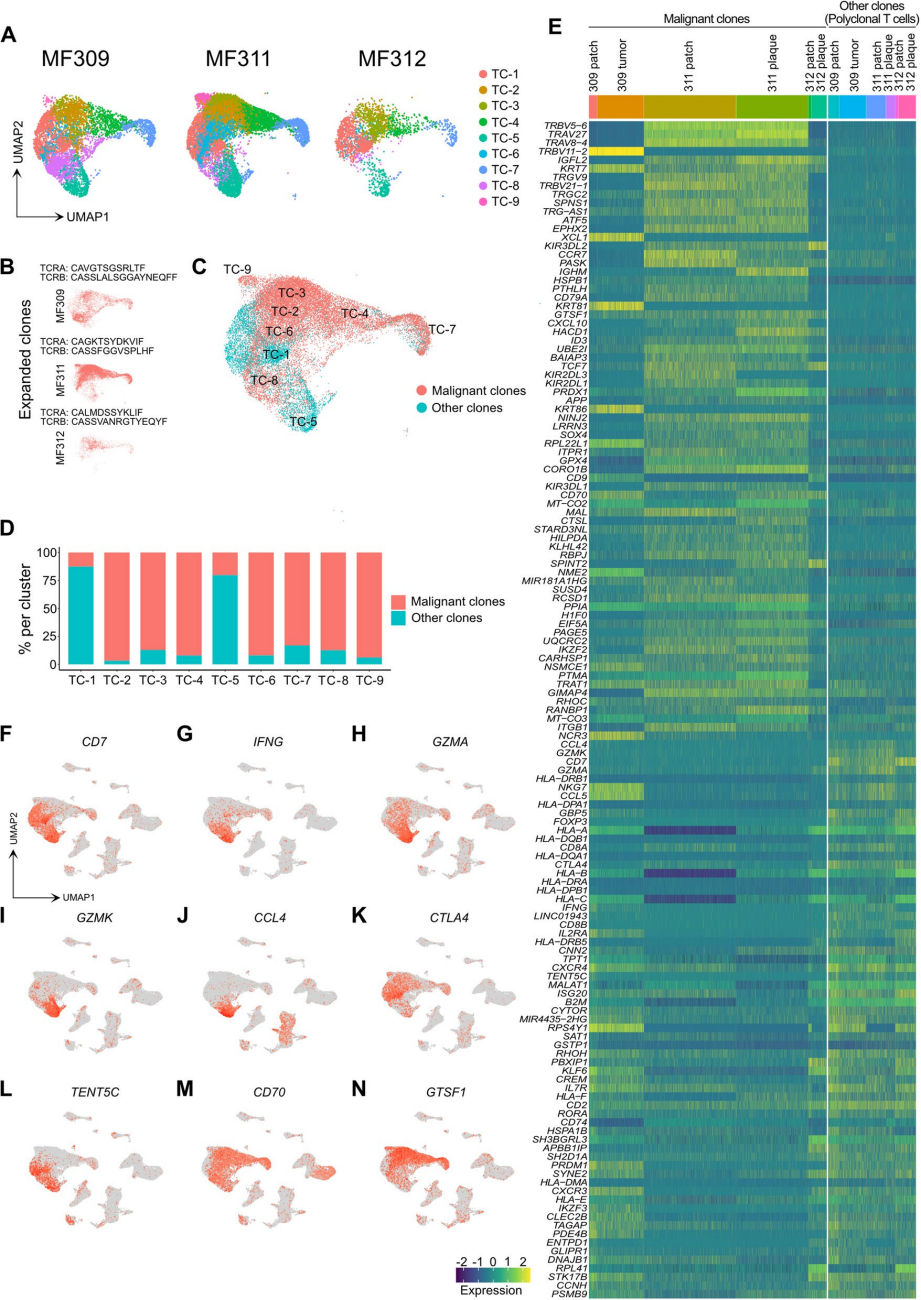
Characteristics of T cell clusters. **A** Separate UMAP plots of T cell clusters from skin samples of patients MF309, MF311 and MF312. **B** UMAP plots of the single top expanded clone (according to the most common α and/or β chain CDR3s aminoacid sequence) per patient. **C** Combined UMAP of T cell clusters colored according to clonality of TCR. Red: Cells with top expanded monoclonal TCR(s) from each sample; turquoise: cells with polyclonal TCR. **D** Percentage of monoclonal and polyclonal populations per T cell cluster. **E** Heat map displaying top differentially expressed genes between expanded clones and polyclonal TCR+ cells, according to the smallest adjusted *p*-value and average log fold change as calculated by logistic regression with Bonferroni correction; upregulation is indicated in yellow, and downregulation in blue/green; gene names are shown on the left. **F-N** Combined feature plots showing expression of selected genes differentially expressed between top expanded clones and the polyclonal infiltrate. Normalized expression level for each cell is color-coded (red) and overlaid onto UMAP plots. TC: T cells; UMAP: Uniform Manifold Approximation and Projection. TCR: T-cell receptor

Both in patch and plaque/tumor lesions, T cells represented the largest fraction of cells (Fig. 1 C, Table S1). Most of the nine individual T-cell clusters were found in all three patients, except for TC-9, that was mainly present in MF311 (Fig. 2 A), characterized by the overexpression of the chemokine receptor *CCR7* (Table S2) that is typically found in recirculating T cells [22]. By combining 5′ scRNA-seq with αβ T-cell receptor (TCR) sequencing, we defined the top expanded clone within each patient (Fig. 2 B). This clone comprised 58.4, 84.0 and 47.6% of all TCR-positive cells in MF309, MF311 and MF312, respectively, (Fig. S2G-H), thus most likely constituting the malignant clone [23]. Clonally expanded cells were primarily found in *CD4+* clusters TC-2, TC-3, TC-4, TC-6, TC-7, TC-8 and TC-9, while TC-1 (helper and regulatory T cells) and TC-5 (cytotoxic T cells) largely consisted of polyclonal T cells (Fig. 2 C-D). To better understand transcriptomic features of putatively malignant (monoclonal) vs. benign (polyclonal) T cells, we calculated differentially expressed genes (DEGs) between these two groups and displayed top DEGs in a heat map (Fig. 2 E). We found considerable inter-patient variability in monoclonal populations, while gene expression in polyclonal cells seemed more homogeneous (Fig. 2 E, Fig. S2I), despite the fact that the latter comprised both *CD4+* and *CD8A+* subsets. Patient-to-patient heterogeneity was also clearly visible when DEGs in monoclonal vs. polyclonal T cells were calculated for each patient separately (Fig. S2J-L). One of the few markers generally downregulated in malignant cells included *CD7* (Fig. 2 F, S2J-L), in line with a malignant phenotype, and as seen in histopathology (Fig. 1 A) [24]. Other genes largely absent in monoclonal cells included the prototypic type 1 cytokine *IFNG*, the cytotoxic molecules *GZMA* and *GZMK*, and the chemokine ligand *CCL4* (Fig. 2 E), which were mostly found in benign *CD8A+* cells (Fig. 2 G-J), in line with cytotoxic T cells. Similarly, lymphoma cells lacked *CTLA4* expression (Fig. 2 E), which was mostly present in regulatory T cells of cluster TC-1 (Fig. 2 K). In contrast, the tumor suppressor gene *TENT5C* [25] was expressed in both *CD8A+* T cells and regulatory T cells (Fig. 2 L). Only a few genes were consistently upregulated in clonally expanded T cells, including *CD70*, a marker of highly activated lymphocytes, and *GTSF1* (gametocyte specific factor 1) (Fig. 2 E, M, N), as previously reported in CTCL [12, 26, 27]. Taken together, we were able to detect a single clone that was strongly expanded in each patient sample, harboring a malignant phenotype. Notably, the dominant clones showed considerable inter-individual transcriptomic heterogeneity.

### *CXCR4, CD69, HSPA1A, ZFP36, IL7R* and *TXNIP* are consistently downregulated in the malignant clone in palpable vs. flat skin lesions

**Fig. 3:**
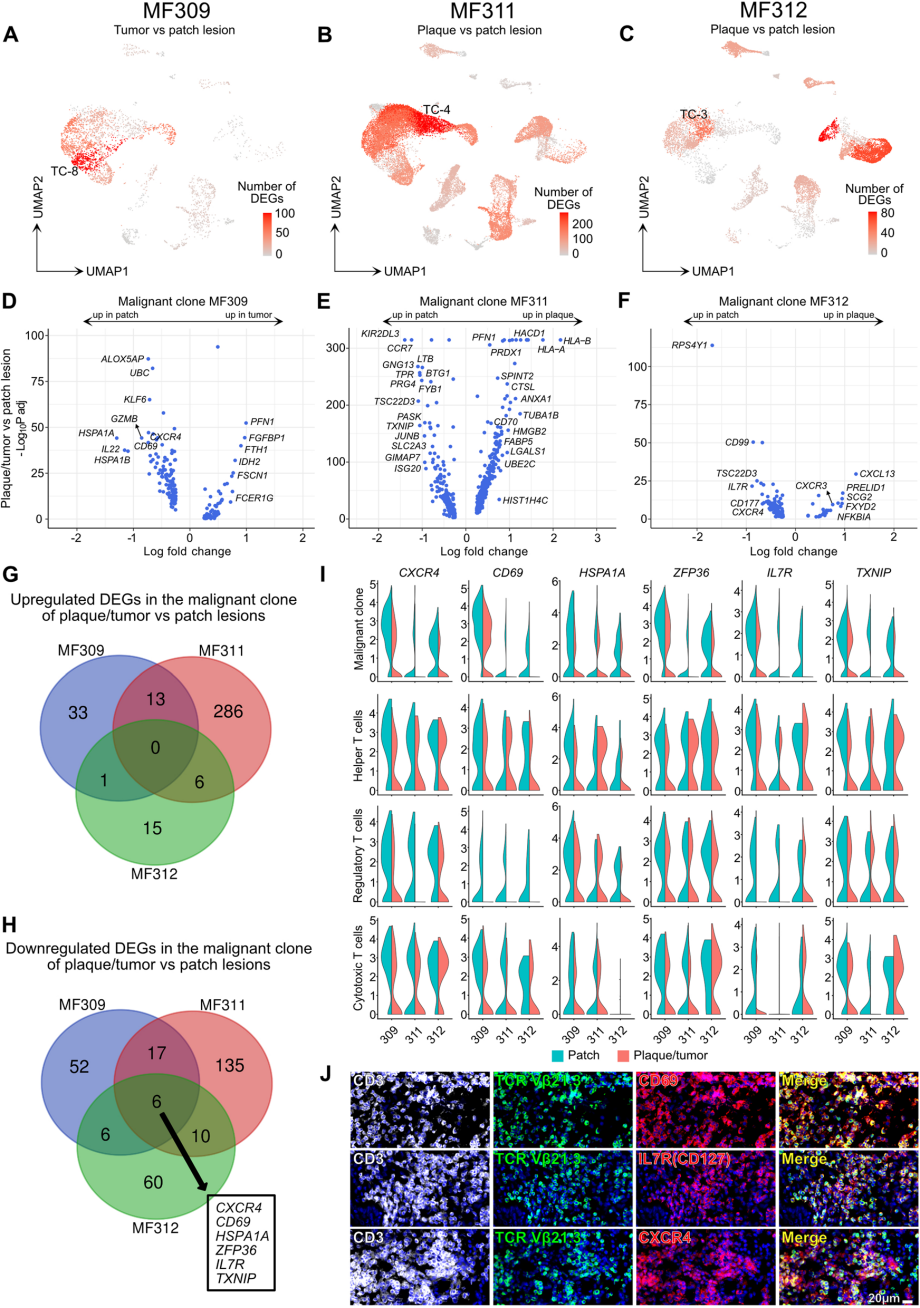
Differential gene expression between plaque/tumor and patch lesions is prominently found in expanded T cell clones. **A-C** Total number of differentially expressed genes (DEGs) comparing plaque/tumor with patch lesions for each cluster, projected onto the respective UMAP plots. Differential gene expression was defined as log fold change >∣0.3∣ and adjusted p-value< 0.05 as calculated by logistic regression and Bonferroni correction. **D-F** Volcano plots of DEGs in malignant cells comparing plaque/tumor and patch lesions. **G-H** Venn diagram of significantly up- or downregulated genes comparing gene expression in the malignant clone of plaque/tumor vs. patch lesions (defined by an adjusted *p* value< 0.05 and a logFCH>|0.3|). **I** Violin plots of T-cell clusters showing distribution of normalized gene expression levels of the six genes consistently downregulated in plaque/tumor (red) vs. patch lesions (turquoise). Malignant clone: top expanded clone. Helper T cells: *CD4+ FOXP3-* cells with polyclonal TCRs. Regulatory T cells: *FOXP3+* cells with polyclonal TCRs. Cytotoxic T cells: *CD8A+ FOXP3-* cells with polyclonal TCRs. **J** Representative immunofluorescence pictures of lesional MF skin (advanced-stage plaque) using a clone-specific antibody (anti-TCRVβ21.3) in order to visualize tumor cells (green) within the CD3+ T cell population (white), confirming co-expression with CD69, CXCR4 and IL7R/CD127 (red); DAPI-stained cell nuclei appear blue; “merge” (yellow) denotes overlay of green and red staining; pictures representative for 3 individual experiments. UMAP: Uniform Manifold Approximation and Projection

To investigate markers of skin lesion progression, we compared patch vs. plaque or tumor lesions for each patient individually, by calculating total numbers of DEGs per cluster (Fig. 3 A-C). Within T cells, three malignant clusters displayed the highest numbers of DEGs (TC-8 in MF309, TC-4 in MF311, and TC-3 in MF312), while benign clusters TC-1 and TC-5 harbored only a few DEGs. When comparing malignant clones in plaque/tumor vs. patch lesions (Fig. 3 D-F, Table S3), we did not find DEGs that were consistently upregulated in all three patients (Fig. 3 G). By contrast, we discovered six genes to be downregulated in all patients in plaque/tumor vs. patch lesions, namely the chemokine receptor *CXCR4*, the skin residency marker *CD69*, the heat shock protein *HSPA1A*, the anti-inflammatory mediator tristetraprolin (i.e. zinc finger protein 36 homolog *ZFP36*), the interleukin-7 receptor *IL7R*, and the thioredoxin-interacting protein *TXNIP* (Fig. 3 H, Table S3). While some of these markers were also downregulated in benign T cell populations including *CD4+* helper T cells, *CD8A+* cytotoxic T cells, and *FOXP3+* regulatory T cells, consistent downregulation across all three patients was only observed in malignant cells (Fig. 3 I, Table S3). Importantly, no single other gene was consistently regulated in benign T cell populations between plaque/tumor and patch lesions in all three patients (Table S3). By using immunohistochemistry, we were able to confirm that malignant cells (as defined by staining of the expanded clone with antibodies specific for the respective T-cell receptor beta chain) co-expressed the cell surface markers CD69, CXCR4, and IL7R (CD127) on a protein level (Fig. 3 J). We also assessed marker expression of lymphoma cells isolated from three patch and five plaque/tumor MF skin lesions by flow cytometry-based cell sorting (Fig. S3A-B), confirming similar decreases in all markers except for *TXNIP*. These data suggest that *CXCR4, CD69, HSPA1A, ZFP36, IL7R* (and possibly *TXNIP)* represent potential markers of disease progression that are common to the malignant clone in the three MF patients investigated.

### Cells of the lymphoma microenvironment only show few consistent transcriptomic changes from patch to plaque/tumor lesions

**Fig. 4:**
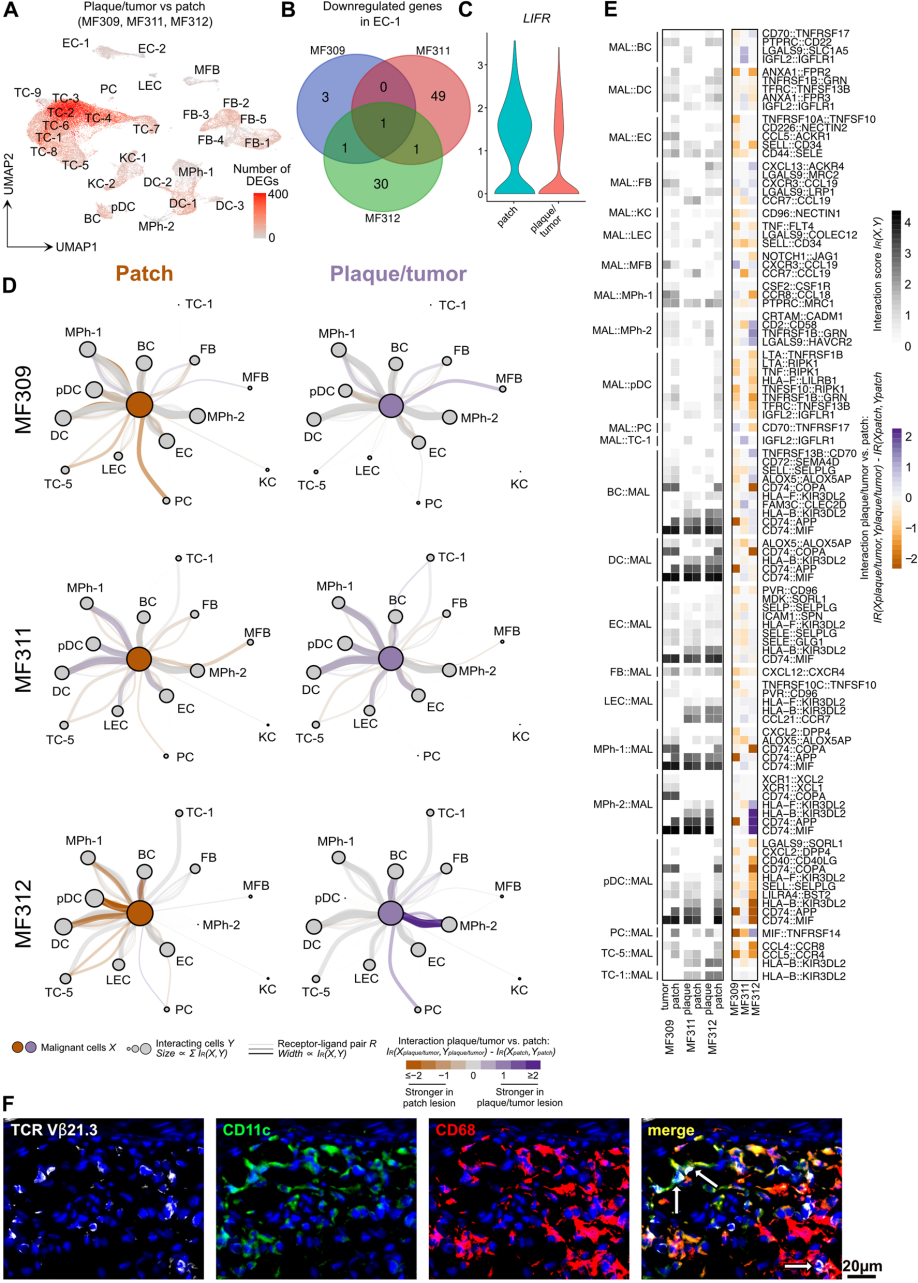
Lack of consistent gene regulation or receptor/ligand interaction in cells of the microenvironment across patients. **A** Total numbers of differentially expressed genes (DEGs) within each cluster comparing palpable (plaque/tumor) with patch lesions in pooled samples, projected onto the UMAP plot. Differential gene expression was defined as log fold change >∣0.25∣ and adjusted *p* < 0.05 as calculated by logistic regression and Bonferroni correction. **B** Venn diagram of significantly downregulated genes comparing the gene expression between plaque/tumor and patch lesions in the endothelial cell cluster 1 EC-1 (adjusted p value< 0.05, logFCH>|0.25|). **C** Violin plots of *LIFR* expression in cluster EC-1 showing the distribution of normalized gene expression levels in pooled cells of patch (turquoise) and plaque/tumor (red) lesions. **D** Graph visualization of putative cell-cell interactions. Interactions were inferred by co-expression of ligand-receptor pairs (*R*) from CellPhoneDB [28] between malignant T cells (*X*) and each cell cluster (*Y*) in the same sample. Edge width is proportional to the interaction score (*I*_*R*_*(X,Y)*), which reports the mean expression of the receptor and ligand in the respective cell clusters. Node size is proportional to the sum of all connected interaction scores. Edge color indicates the difference of interaction scores between patch and plaque/tumor lesions of the same patient. Only receptor-ligand pairs with a significant interaction score in at least one sample are shown (FDR-adjusted empirical *p*-value <= 0.05, see *Methods*). **E** Heat map visualization of cell-cell interaction scores for each indicated receptor-ligand and cell type pair in each sample (left) or the difference between interaction scores in plaque/tumor and patch lesions (right). Interaction scores (*I*_*R*_*(X,Y)*) were inferred by co-expression of ligand-receptor pairs (*R*) from CellPhoneDB [28] between malignant T cells (*X*) and each cell cluster (*Y*) in the same sample. Only receptor-ligand pairs with a significant interaction score in at least one sample are shown (FDR-adjusted empirical *p*-value <= 0.05, see *Methods*). Dendritic cells DC-1, DC-2, DC-3, keratinocytes KC-1 and KC-2, fibroblasts FB-1 to FB-5, and malignant T cell clusters were pooled for analyses. **F** Representative immunofluorescence stainings of lesional MF skin (plaque) using a clone-specific anti-TCRVβ21.3 antibody to visualize cells of the expanded malignant clone, to demonstrate their vicinity (white arrows) to CD11c + CD68+ DCs and CD11c- CD68+ macrophages; DAPI-stained cell nuclei appear blue. Pictures are representative of three independent experiments. TC T cells; BC B cells; KC keratinocytes; FB fibroblasts; DC dendritic cells; MPh macrophages; MFB myofibroblasts; EC endothelial cells; LEC lymphoendothelial cells; pDC plasmacytoid dendritic cells; PC plasma cells

Besides the malignant clone, benign infiltrating immune cells as well as non-leukocytes are assumed to be involved in the advancement of cutaneous lymphoma lesions [9]. However, DEGs between plaque/tumor vs. patch lesions were generally low in non-malignant cells (Fig. 4 A, Table S4), with single patients dominating the transcriptomic pattern of certain cell clusters (Fig. 3 A-C). When comparing DEGs between plaque/tumor and patch lesions of non-malignant cells that were mutually regulated in each patient, we only found *LIFR* (leukemia inhibitory factor receptor or CD118) in the EC-1 cluster to be downregulated (Fig. 4 B-C), the receptor for leukemia inhibitory factor (LIF) previously characterized as a tumor suppressor gene [29]. Functional differences in the relationship of the malignant clone with its microenvironment might, however, also occur on the level of receptor-ligand pairings where not always the same partner is affected across patients. Thus, we also assessed putative interactions between the malignant clone and non-malignant cells, as inferred by co-expression of ligand-receptor pairs (*R*) from CellPhoneDB [28] within each sample (Fig. 4 D-E, Table S5). Across patients, most ligand/receptor pairs were found for myeloid cells (dendritic cells, macrophages), followed by B cells and endothelial cells, while interaction scores of malignant cells with fibroblasts and keratinocytes were generally low (Fig. 4 D). In line with tumor cell - myeloid cell interactions, immunofluorescence stainings confirmed the close vicinity of the expanded clone (as defined by specific TCR expression) with CD11c + CD68+ dendritic cells and CD11c- CD68+ macrophages within MF lesional skin, both in plaque (Fig. 4 F) and patch lesions (data not shown), suggesting cell-to-cell contact between these populations. While some receptor/ligand pairs were present at high levels across all samples, such as *CD74 - MIF* on myeloid cells/malignant T cells, expression patterns were heterogeneous between patients (Fig. 4 E). The few receptor/ligand pairs that were consistently different between plaque/tumor vs. patch lesions included *SELL - CD34* (malignant cells/lymphatic endothelial cells), *CXCL12 - CXCR4* (fibroblasts/malignant cells), and *CCL5 - CCR4* (cytotoxic T cells/malignant cells), all of which were downregulated with skin lesion progression (Fig. 4 E, Table S5). Most detected pairs, however, showed divergent regulation with lesion progression, further supporting the concept of patient-to-patient heterogeneity.

### Corroboration of regulated genes in a case of γδ MF

**Fig. 5:**
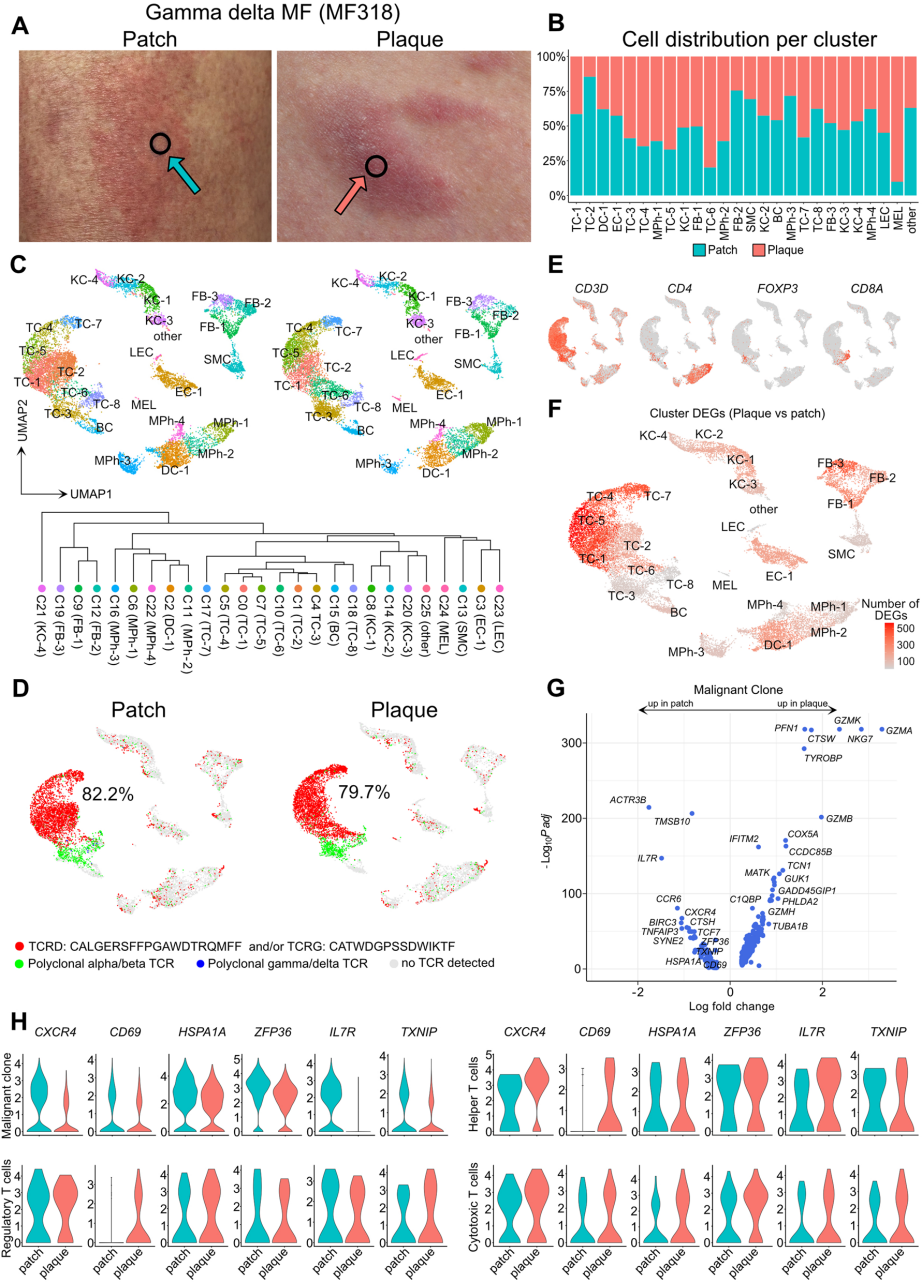
Gene expression in patch and plaque lesions of a γδ TCR+ MF patient. **A** Pictures of patch and plaque lesions; black circles indicate biopsy location. **B** Relative distribution of cells within individual clusters in patch vs. plaque lesions. **C** UMAP of 22,051 cells integrated from two skin biopsies according to similarity of their transcriptome, resulting in 26 different color-coded clusters (C0-C25), split according to tissue of origin. **D** UMAP plots of patch and plaque samples colored according to most common monoclonal γδ TCR (red), polyclonal αβ (green) or polyclonal γδ TCRs (blue), and cells without detectable TCR (grey). Percentages denote frequencies of malignant cells among all TCR+ cells per plot. **E** Feature plots showing expression of selected T cell marker genes. Normalized expression level for each cell is color-coded (red) and overlaid onto UMAP plots. **F** Total numbers of differentially expressed genes (DEGs) within each cluster comparing plaque with patch lesions, projected onto an UMAP plot. Differential gene expression was defined as log fold change >∣0.25∣ and adjusted p < 0.05 as calculated by logistic regression and Bonferroni correction. **G** Volcano plot of DEGs of the malignant clone between patch and plaque lesions. **H** Violin plots of T cell clusters showing distribution of normalized gene expression levels of the six uniformly differentially expressed genes in patch (turquoise) and plaque (red) lesions. Malignant clone: top expanded γδ clone. Helper T cells: *CD4+ FOXP3-* cells with polyclonal TCRs. Regulatory T cells: *FOXP3+* cells with polyclonal TCRs. Cytotoxic T cells: *CD8A+ FOXP3-* cells with polyclonal TCRs. UMAP: Uniform Manifold Approximation and Projection

In order to investigate whether our findings from three classic MF patients could be a more general mechanism among other forms of MF, we performed scRNA-seq in skin samples from a patient newly diagnosed with MF of a TCR γδ phenotype (MF318), who also presented with longstanding patches and newly developed plaques (Table 1, Fig. 5 A). Except for a minor population of melanocytes, both patch and plaque lesions harbored comparable cell clusters and numbers (Fig. 5 B-C, Table S6), with an abundance of TCR γδ positive T cells, that derived from a single clone (Fig. 5 D, labelled in red). By contrast, polyclonal T cells were of an αβ TCR phenotype (Fig. 5 D, labelled in green), containing *CD4+* helper T cells, *CD8A+* cytotoxic T cells, and *FOXP3+* regulatory T cells (Fig. 5 E). Polyclonal γδ T cells were essentially absent (Fig. 5 D, labeled in blue). When calculating DEGs in plaque vs. patch lesions, we again found most DEGs to be present in monoclonal γδ T cell clusters (Fig. 5 F, Table S6-S7), that included cytotoxic markers such as *GZMA, NKG7, GZMK, GZMB, CTSW*, *TYROBP* (DAP12), and *GZMH*, but also the coiled-coil domain containing 85B (*CCDC85B*) previously associated with cancer proliferation and invasion [30], and the actin-binding *PFN1* (Profilin-1) (Fig. 5 G, Table S6). Similar to abovementioned αβ MF patients, the malignant clone in this γδ MF patient also showed significant decreases in *CXCR4, CD69, HSPA1A, ZFP36, IL7R* and *TXNIP* in plaque vs. patch lesions (Fig. 5 H, Table S6). Importantly, a decrease in these markers was absent in polyclonal T cell populations, namely *CD4+* helper T cells, *CD8A+* cytotoxic T cells, and *FOXP3+* regulatory T cells, which showed relatively stable expression or even trends of increase in plaques (Fig. 5 H, Table S6). Other top downregulated markers in clonal cells included the actin-binding markers *TMSB10* and *ACTR3B*, the CCL20 chemokine receptor *CCR6*, the apoptosis inhibitor *BIRC3*, cathepsin H (*CTSH)*, and the TNF-induced anti-inflammatory mediator *TNFAIP3* (Fig. 5 G). In non-T cells, we found highest numbers of DEGs in fibroblasts (Fig. 5 F, Table S7). The FB-3 cluster showed upregulation of genes involved in collagen biosynthesis and extracellular matrix organization (various *COL* genes, *P4HB, ADAMTS2, LOXL2, LOXL1*). In line with the concept of downregulated Th1 responses upon disease progression [9], we found decreased levels of the type-1-associated chemokines *CXCL9* and *CXCL10* in several cell populations in plaques vs. patches, including DC-1, EC-1, FB-1, MPh-2, and MPh-4 (Table S7). In sum, malignant γδ MF cells showed identical regulation of *CXCR4, CD69, HSPA1A, ZFP36, IL7R* and *TXNIP* as found in αβ MF clones, suggesting their involvement in disease progression across MF subtypes.

### *CD69, HSPA1A* and *ZFP36* are further elevated in patches of early-stage MF

**Fig. 6:**
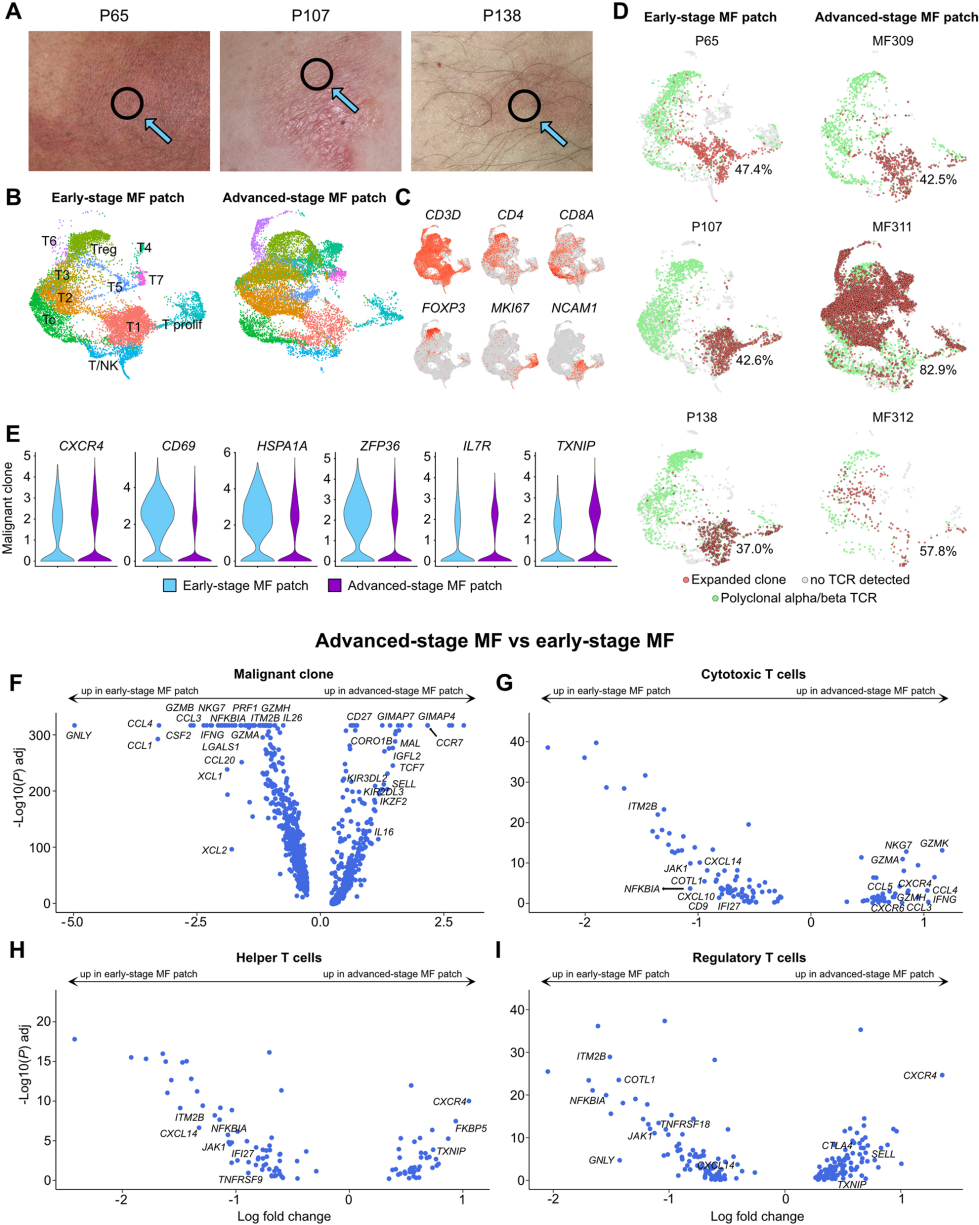
Comparison of patch lesions from advanced-stage vs. early-stage disease. **A** Pictures of patch lesions from 3 patients with longstanding, early-stage MF; black circles indicate biopsy location. **B** UMAP of 20,975 cells from three early-stage (P65, P107, P138) and three advanced-stage MF patches (MF309, MF311, MF312) according to similarity of their transcriptome. T-cell harboring clusters from Fig. S4 (clusters TC-1 to TC-8, Prolif and Treg, as depicted in Fig. S4A) were used for reclustering, resulting in 11 different color-coded clusters, split according to tissue of origin. **C** Feature plots showing expression of selected T and NK cell marker genes. Normalized expression level for each cell is color-coded (red) and overlaid onto UMAP plots. **D** UMAP plots of individual patients colored according to most common monoclonal (red) and polyclonal (green) αβ TCR; cells without TCR are displayed in grey. Percentages denote frequencies of malignant cells among all TCR+ cells for each plot. **E** Violin plots showing distribution of normalized gene expression levels in the top expanded αβ TCR clone in early (light blue) vs. advanced-stage MF (purple). **F-I** Volcano plot showing differentially expressed genes (DEGs) of the malignant clone, as well as polyclonal helper, cytotoxic and regulatory T cells between advanced and early MF patch lesions. Differential gene expression was defined as log fold change >∣0.25∣ calculated by logistic regression and Bonferroni correction. Regulatory T cells: *FOXP3+* cells with polyclonal TCRs. Cytotoxic T cells: *CD8A+ FOXP3-* cells with polyclonal TCRs. Helper T cells: *CD4+ FOXP3-* cells with polyclonal TCRs. UMAP: Uniform Manifold Approximation and Projection

As above-mentioned analyses were all performed in patients with advanced-stage disease, we next wanted to compare our data with skin from patients with longstanding, early-stage disease, who had been showing an indolent clinical course over years to decades (Fig. 6 A, Table 1). T-cell subsets contained generally comparable cell counts in early-stage MF (9839 cells from 3 patients) and in patch lesions from MF patients with advanced-stage disease (11,136 cells from 3 patients), and both showed substantial numbers of proliferating cells (Fig. 6 B-C, Table S8). Interestingly, early-stage MF showed distribution of expanded clones primarily in clusters T1, T/NK, and the proliferating cluster, in contrast to malignant cells from patches of advanced-stage disease, which spread over several additional clusters in a more inconsistent fashion (Fig. 6 D). In line with decreasing levels in advanced plaque/tumor lesions, the markers *CD69, HSPA1A* and *ZFP36* showed significantly higher expression levels in patches of early-stage MF in comparison to patches from advanced-stage MF (Fig. 6 E), while other T subsets (helper T cells, cytotoxic T cells, regulatory T cells) again did not consistently harbor such differences (Table S8). Genes that were present at significantly higher levels in malignant cells of patches from advanced vs. early-stage MF included the lymph node homing markers *CCR7*, *SELL* and *CD27* [31], the CTCL markers *IGFL2* and *KIR3DL2* [32, 33], the helper T cell growth factor *IL16* that has previously been implicated in recruitment of malignant cells to MF lesions [34], and the lymphocyte developmental marker *IKZF2* [35] (Fig. 6 F). Markers predominantly found in tumor cells of early-stage MF lesions included cytotoxic molecules such as *GNLY, GZMA, GZMB* and *GZMH,* as well as a broad array of inflammatory cytokines/chemokines such *IFNG*, *CCL4, CCL1, CSF2, CCL3, CCL20, IL26, XCL1,* and *XCL2* (Fig. 6 F)*.* In benign cells, by contrast, cytotoxic and associated mediators (*GZMK, IFNG, GZMH, NKG7, GZMA*) were upregulated in cytotoxic T cells of advanced-stage, and not early-stage patches (Fig. 6 G). Early-stage lesions, however, exerted increased expression of the NF-kB inhibitor *NFKBIA* and the anti-angiogenic chemokine *CXCL14* in helper, cytotoxic and regulatory T cells (Fig. 6 G-I). Among non-T cells (Fig. S4A-B), we found increased levels of the Th2-associated chemokine *CCL17* in macrophages and *LAMP3+* mature DC-3, and concomitant decreases in the Th1-associated markers *CXCL9* and *CXCL10* in dendritic cells and macrophages of advanced-stage lesions (Table S9), corroborating the concept of a shift in Th2/Th1-associated markers during disease progression in MF [9].

### Phenotypic changes in follow-up samples during disease exacerbation

**Fig. 7:**
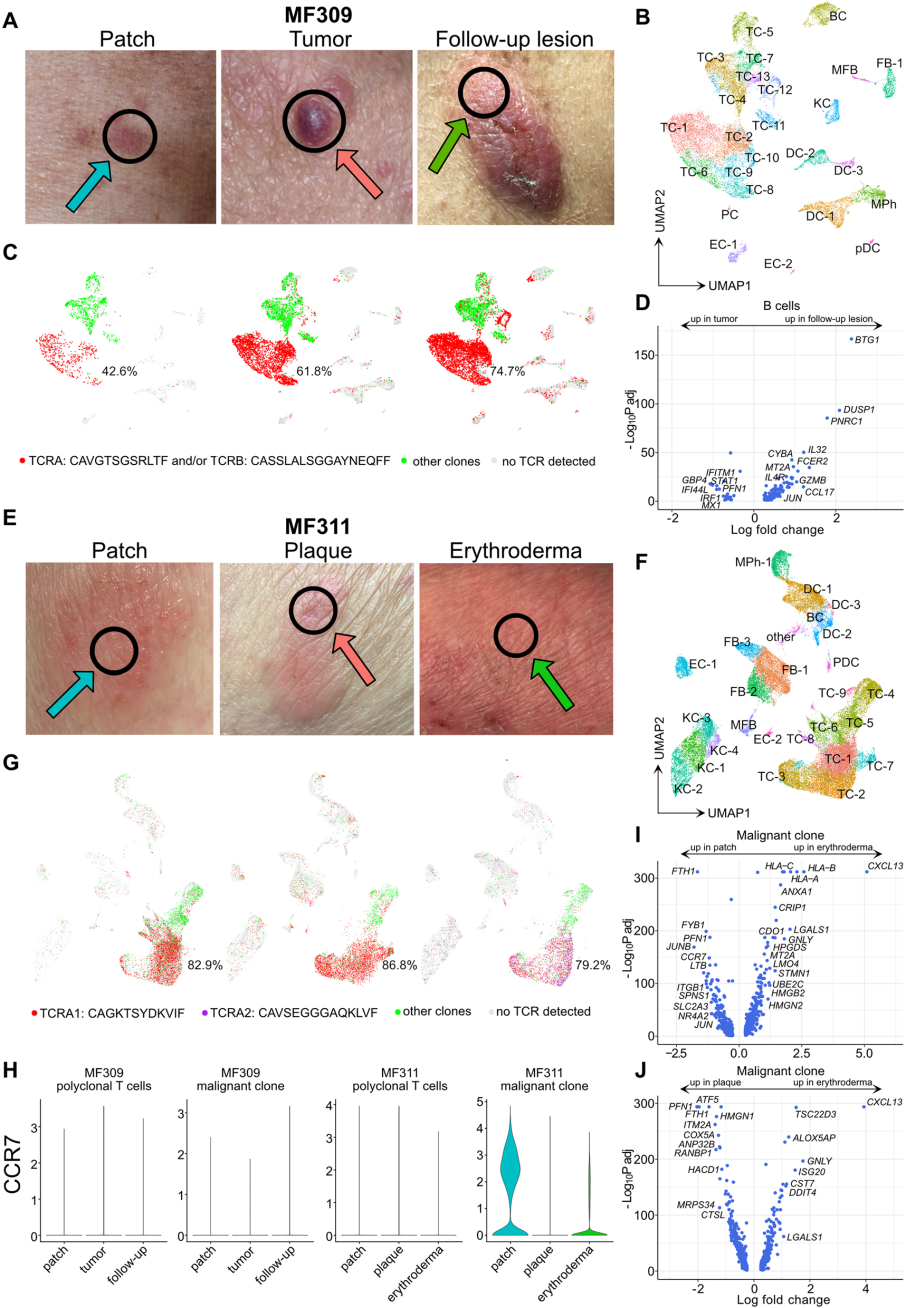
Characterization of follow-up lesions during disease exacerbation. **A** Pictures of MF lesions of patient MF309 showing initial flat (patch) and palpable (tumor), as well as follow-up (ulcerated tumor) lesions. Black circles indicate the location where the respective biopsy was taken from. **B** UMAP of 20,944 cells integrated from these three MF309 skin biopsies according to similarity of their transcriptome, resulting in 25 different color-coded clusters. **C** UMAP plots of samples colored according to most common monoclonal TCR (red), polyclonal αβ TCRs (green) and cells without detectable TCR (grey). Percentages denote frequencies of malignant cells among all TCR+ cells for each plot. **D** Volcano plot showing differentially expressed genes in B cells in follow-up vs. tumor lesions. **E** Pictures of MF lesions of patient MF311 showing initial patch and plaque, as well as follow-up lesions (erythroderma). Black circles indicate the location where the respective biopsy was taken from. **F** UMAP of 40,020 cells integrated from three MF311 skin biopsies according to similarity of their transcriptome, resulting in 26 different color-coded clusters. **G** UMAP plots of samples colored according to most common monoclonal TCRs TCRA1 (red), TCRA2 (purple), polyclonal αβ TCRs (green) and cells without TCR detected (grey). Percentages denote frequencies of malignant cells among all TCR+ cells for each plot. **H** Violin plots showing distribution of normalized *CCR7* gene expression levels of benign and malignant T cells in the respective skin lesions. **I-J** Volcano plots of differentially expressed genes within the malignant clone of MF311 comparing follow-up with patch and plaque lesions, respectively. TC T cells; BC B cells; KC keratinocytes; FB fibroblasts; DC dendritic cells; MPh macrophages; MFB myofibroblasts; EC endothelial cells; pDC plasmacytoid dendritic cells

To better understand the dynamics of disease progression in MF, we followed patients over time and performed new biopsies upon phenotypic change or treatment response, and integrated the new data sets with the existing scRNA-seq data, separately for each individual. After 4 cycles of brentuximab vedotin resulting in a complete response and ongoing treatment with extracorporeal photopheresis (ECP), patient MF309 experienced progressive disease with generalized ulcerating tumors 9 months after initial sampling (Fig. 7 A). The single malignant clone (Fig. 7 B-C, Tables S10-S12) maintained decreased expression of *CXCR4, CD69, HSPA1A,* and *ZFP36* in the follow-up lesion*,* while *IL7R* and *TXNIP* showed a trend of increase towards levels found in the initial patch lesion (Table S10, Fig. S5A). Malignant cells of the follow-up lesion were characterized by increases in the pro-tumorigenic mediator *LTB* [36], as well as NK-associated receptor *KLRC1* (NKG2) and *CD74*, both known to promote T cell survival, while cytotoxic chemokines *XCL1* and *XCL2* were decreased (Table S10). Among non-T cells, there was an increase in B cells with advancement of disease, which were virtually absent in patch lesions (Fig. 7 B-C). In the ulcerated tumor, B cells showed increased markers associated with type-2 responses such as *CCL17, IL4R*, and decreases in interferon-stimulated genes (*IFI44L, STAT1, IFITM1, MX1, IRF1,* Fig. 7 D, Table S12). Together with decreases in *IFNG* expression in the benign proliferating TC-11 cluster (Table S12), these findings were consistent with the established concept of decreasing type-1 and increasing type-2 skewing in progressing MF lesions [9].

In contrast to patient MF309, patient MF311 did not develop tumors, but erythroderma 7 months after initial sampling, despite combination treatment with ECP, chlorambucil and systemic glucocorticoids (Fig. 7 E). This follow-up lesion showed absence of three T cell clusters, namely clusters TC-6 and TC-8 (found in the initial patch lesions) and TC-7 (found only in the plaque lesion; Fig. 7 F-G). Both TC-6 and TC-8 were rich in *CCR7* expression (Table S13). CCR7 is a chemokine receptor initially described in naïve and T memory stem cells [37] and a characteristic marker for recirculating T cells that are typically found in MF lesions with ill-defined as opposed to sharply demarcated borders [22], consistent with the clinical (Fig. 7 A, E) and molecular phenotype (Fig. 7 H) of malignant clones in our patients.

TC-7, primarily present in the plaque lesion, was characterized by increased levels of *PFN1* (Table S13), similar to what we observed in γδ MF plaques (Fig. 5 G), and showed elevated levels of the T-cell activation marker *ITM2A* and the cutaneous lymphoma marker *IGFL2* [32] (Table S13). Overall frequencies of monoclonal vs. polyclonal T cells remained relatively stable over time, and the majority of malignant cells expressed the TCR-α chain *CAGKTSYDKVIF* (Fig. 7 G). However, a second TCR-α chain *CAVSEGGGAQKLVF* increased from 6.8 and 1.2% in initial patch and plaque lesions, respectively, to 40.1% of TCR-α + malignant clones in erythrodermic lesions (Fig. 7 G). Nevertheless, both TCR-α chains (labelled TCRA1 in red, and TCRA2 in purple in Fig. 7 G) showed pairing of the same TCR-β chain *CASSFGGVSPLHF* (data not shown), suggesting that both TCRA1+ and TCRA2+ cells represent the same clone, albeit with differing levels of allelic exclusion [38]. In line, there was only a limited number of DEGs present at a low log fold change when comparing TCRA1 with TCRA2 clones in the follow-up lesion (Table S13, “TRA_CAG vs. CAV”). Low expression of *CXCR4, CD69, ZFP36* and *TXNIP* was maintained upon disease exacerbation, while *IL7R* and *HSPA1A* reverted to the levels detected in initial patch lesions (Fig. S5B). In erythroderma, malignant cells showed strong increases in *CXCL13* compared to both patch and plaque lesions (Fig. 7 I-J, Table S13), previously described to be highly upregulated in Sézary cells [39]. Among benign cells, there was a shift towards a more type-2-biased immune microenvironment, as reflected by increases in *CCL17* and *CCL18* expression in myeloid cells, and decreases in the type-1-associated chemokines *CXCL9* and *CXCL10* in myeloid cells, fibroblasts and endothelial cells (Tables S14-S15). Increased inflammatory keratinocyte responses were reflected by elevated levels of inflammatory keratins *KRT6A* and *KRT16* (Tables S14-S15). Taken together, disease exacerbation was associated with increased type-2 responses in both patients, while initial overexpression of *CCR7* preceded erythroderma, but not tumor formation.

### Downregulation of *CXCR4, CD69, HSPA1A, ZFP36, IL7R* and *TXNIP* is reverted in malignant clones of clinically unaffected skin of MF patients

**Fig. 8:**
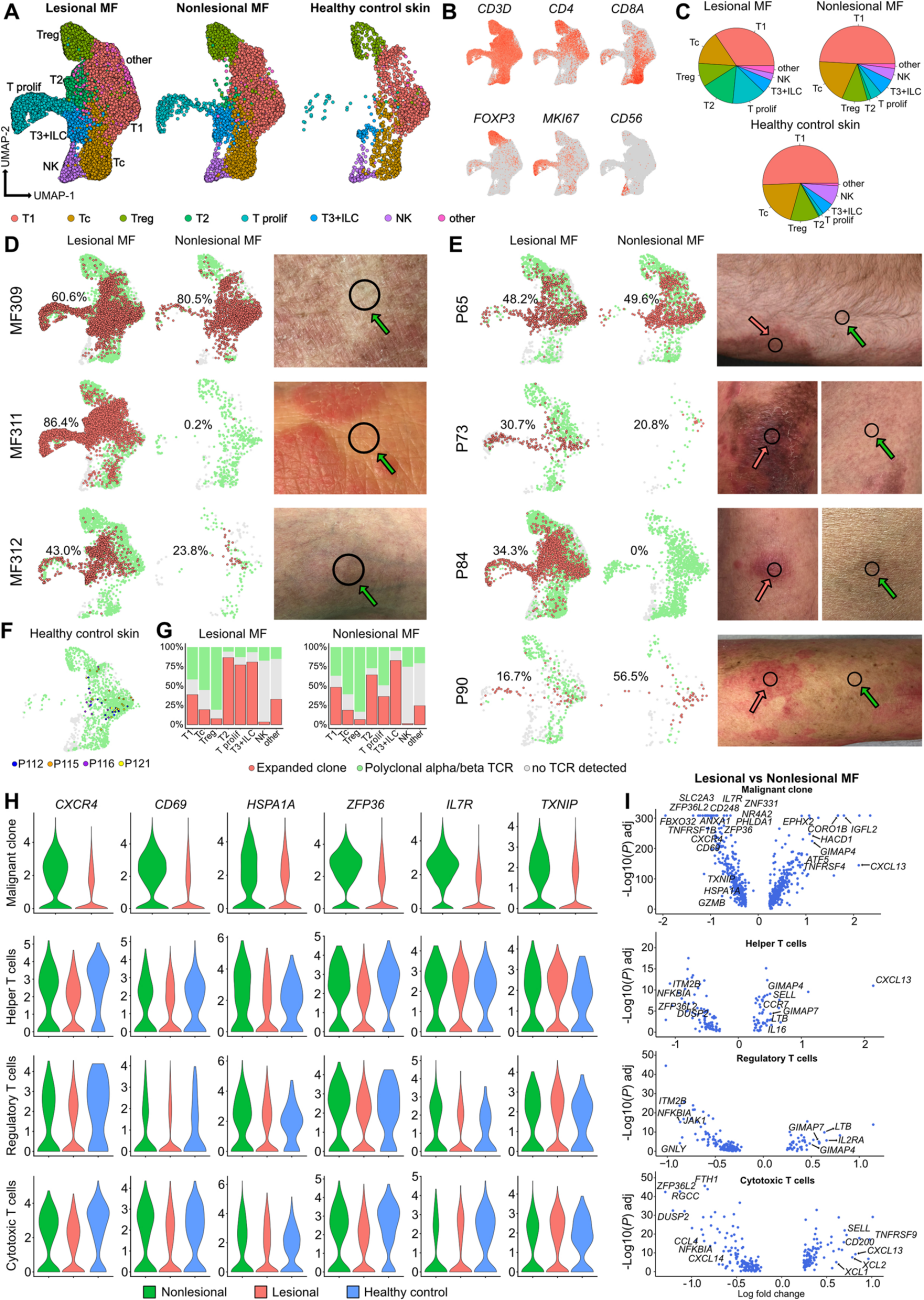
Comparison of lesional with nonlesional MF and healthy control skin. **A** UMAP of 32,958 subclustered cells from lymphocyte clusters TC-1, TC-2, TC-3/NK and TC-4 of Fig. S8, integrated from 7 MF patients with lesional and nonlesional biopsies, and 4 healthy control samples, according to similarity of their transcriptome, resulting in 8 different color-coded clusters, split according to tissue of origin. **B** Feature plots showing expression of selected T cell and NK cell marker genes. Normalized expression level for each cell is color-coded (red) and overlaid onto UMAP plots. **C** Frequencies of cell clusters within groups. **D-E** UMAP plots of individual patients colored according to most common monoclonal (red) and polyclonal (green) αβ TCR; cells without TCR are displayed in grey; photographs of biopsy sites for nonlesional (green arrow) and lesional (red arrow) skin. Percentages denote frequencies of malignant cells among all TCR+ cells for each plot. **F** UMAP of four healthy control biopsies; the single most frequent clone for each sample is depicted in blue (P112), orange (P115), purple (P116) or yellow (P121); all other TCR+ cells are shown in green, and cells without detectable TCR in grey. **G** Distribution of the malignant clone (red), polyclonal TCR+ cells (green), and cells without detectable TCR, as percentages for each cluster in lesional and nonlesional biopsies. **H** Violin plots showing distribution of normalized gene expression levels in nonlesional (green), lesional (red) and healthy control skin (blue) for the top expanded αβ TCR clone, as well as helper T cells, regulatory T cells, and cytotoxic T cells. **I** Volcano plot showing differentially expressed genes (DEGs) of the malignant clone, as well as polyclonal helper, cytotoxic and regulatory T cells between lesional and nonlesional MF biopsies. Differential gene expression was defined as log fold change >∣0.25∣ as calculated by logistic regression and Bonferroni correction. Regulatory T cells: *FOXP3+* cells with polyclonal TCRs. Cytotoxic T cells: *CD8A+ FOXP3-* cells with polyclonal TCRs. Helper T cells: *CD4+ FOXP3-* cells with polyclonal TCRs. UMAP: Uniform Manifold Approximation and Projection

We also followed patient MF312, who received topical chlormethine hydrochloride 160 μg/g gel formulation QD. After 5 weeks of treatment, several of his MF lesions had resolved, and we took a biopsy from such a treated lesion (Fig. S6A-B, Table S16). Despite complete clinical clearance, general decreases in inflammatory mediators (Tables S17-S18), and a substantial reduction in overall T-cell counts, we were surprised to still find 20.6% of all TCR+ cells to be of the malignant clone (labelled in red in Fig. S6C), harboring the same TCR-α chain *CALMDSSYKLIF* as observed in the initial patch and plaque lesion. To assess whether the presence of a malignant clone is a general phenomenon in clinically unaffected skin of MF patients, we profiled lesional and nonlesional skin of seven patients (from patients MF309, MF311 and MF312, as well as additional patients P65, P73, P84 and P90), and compared results to four healthy control individuals (Fig. 8, Fig. S7-S8, Table 1). While clusters containing CD4+ helper T cells (“T1”), CD8+ cytotoxic T cells (“Tc”), FOXP3+ regulatory T cells (Treg), NK cells („NK”) and a small group of „T3 “admixed with *CD3D- KLRD1- KLRB1+ XCL1+* innate lymphoid cells [40] (ILC, Fig. S7A-D) remained relatively stable across all three groups, we noticed a decrease in proliferating cells („T prolif”) in nonlesional MF, with almost absence in healthy control skin (Fig. 8 A-C, Table S19). Similarly, a population “T2” of *IGFL2*+ *KIR3DL2*+ cells was present at substantial levels only in lesional, but not in nonlesional MF or healthy control skin (Fig. 8 A, C, Fig. S7E-F). We found considerable numbers of malignant cells to be present in five out of seven nonlesional MF samples, with frequencies ranging from 20.8 to 80.5% of all TCR+ cells (Fig. 8 D-E). In contrast to MF, top expanded clone frequencies in the four healthy control samples were as low as 1.7 to 7.0% among respective TCR+ cells (Fig. 8 F), consistent with an overall polyclonal T cell pattern. Frequencies of malignant vs. polyclonal cells per cluster were largely comparable between lesional and nonlesional samples, except for decreases in malignant proliferating cells in nonlesional skin (Fig. 8 G). When assessing the *CXCR4, CD69, HSPA1A, ZFP36, IL7R* and *TXNIP* gene panel, we found levels of all 6 markers to be increased in nonlesional vs. lesional MF in malignant cells, but not in other lymphoid cell subsets (helper T cells, regulatory T cells, cytotoxic T cells, and NK cells; Fig. 8 H, Fig. S7G, Table S19). Numbers of ILCs other than NK cells (Fig. S7A-D) were too small to conduct meaningful calculations. Most DEGs between lesional and nonlesional MF were found in cells of the malignant clone (Fig. 8 I, Table S19), with upregulation of *CXCL13, IGFL2, CORO1B, GIMAP4, EPHX2, HACD1,* and *ATF5* in lesional vs. nonlesional MF (Fig. 8 I). *CXCL13* was also strongly upregulated in helper and cytotoxic, but not regulatory T cells (Fig. 8 I). Genes significantly upregulated in helper T cells included the central memory markers *SELL* and *CCR7,* the nucleotide-binding *GIMAP7* and *GIMAP4,* lymphotoxin beta *LTB* and the interleukin *IL16*. Lesional regulatory T cells were characterized by increased levels of *IL2RA, LTB, GIMAP7, GIMAP4*, but decreases in granulysin *GNLY* (Fig. 8 I). Lesional cytotoxic T cells showed upregulation of the co-stimulatory molecule *TNFRSF9* (CD137)*,* the cytotoxic cytokines *XCL2* and *XCL1,* as well as *SELL*, and decreases in the macrophage inflammatory protein *CCL4*, a chemokine that was also downregulated in NK cells (Fig. 8 I, S7H). When comparing nonlesional MF with healthy control samples, we found cytotoxicity-associated molecules (*GZMA, GZMK, NKG7, GZMH, GZMB, IFNG, CCL5*) still being upregulated in cytotoxic T cells (Fig. S7I, Table S20), while other cell types only showed few regulated genes, such as elevated *CTLA4* in helper T cells (Fig. S7I). These data suggest some maintained cytotoxic activity in CD8+ cytotoxic T cells of nonlesional MF skin. Among non-T cells, we found decreased frequencies of B cells in nonlesional vs. lesional MF, being essentially absent in healthy control skin (Fig. S8A-C, Table S21). Their continuous increase from early to late-stage MF might suggest a role during disease progression (Fig. S8D). B cells in lesional MF displayed increased levels of type 2-associated chemokines *CCL17* and *CCL18*, *LTB*, and *TMSB4X* (Thymosin beta-4) compared to nonlesional MF (Table S22). Other cell types essentially absent in healthy control samples included proliferating fibroblasts FB-6, *KRT16*+ inflammatory keratinocytes KC-6, and *LAMP3+* mature DC-3 (Fig. S8C). Compared to healthy control skin, nonlesional MF still harbored increased levels of type-2 associated chemokines such as *CCL13* and *CCL18* in macrophages, type-1 associated chemokines *CXCL9* and *CXCL10* in dendritic cells, as well as activation markers such as *MX1* and *S100A8* in keratinocytes (Fig. S8E-F, Table S23), suggesting some retained inflammatory environment, despite the absence of clinically visible MF involvement.

## Discussion

Using scRNA-seq combined with αβ and γδ TCR sequencing, we identified a panel of markers that were consistently downregulated in the malignant clone of progressing MF skin lesions. While usual clinical staging of MF (IA-IVB) does not discriminate between patches or plaques, there is a significant difference documented regarding survival rates of patients with early-stage disease presenting with patches only (TNM stage T1a/T2a) compared to those with patches and plaques (TNM stage T1b/T2b) [41]. Our in-depth analysis of patients with advanced-stage disease revealed differences between such flat (patch) and palpable (plaque/tumor) lesion types which might reflect mechanisms contributing to disease progression and impact on overall prognosis. Consistently, the markers that we identified (*CXCR4, CD69, HSPA1A, ZFP36, IL7R* and *TXNIP*) have all been previously described to be either involved in skin homing, cell growth or cancer development. The chemokine receptor CXCR4 is widely expressed on various cell types, and binding of its ligand CXCL12 (stromal cell-derived factor-1, SDF-1) triggers multiple signaling pathways involved in cell migration, hematopoiesis, bone marrow retention, and tissue homing [42, 43]. Other CXCR4 ligands include ubiquitin, which can act as an anti-inflammatory immune modulator and endogenous opponent of proinflammatory DAMPs [44], and the inflammatory cytokine macrophage migration inhibitory factor (MIF) [42]. CXCR4 has been found in MF cells and tumor-infiltrating lymphocytes [45], but reports on its expression levels in conjunction with distinct disease stages have been contradictory [46–48]. Importantly, the contribution of CXCR4 to skin homing and retention has been shown for Sézary cells [49] and skin-residing acute myeloid leukemia (AML) cells in patients with cutaneous metastases [50]. Thus, decreased expression of *CXCR4* in malignant MF cells upon disease progression might facilitate lymphoma cell mobility, or even dissemination. This effect is likely supported by concomitant decreases in *CD69*, a marker whose expression is associated with prolonged tissue retention of T_RM_ in the skin [51]. While CD69 has initially been described as an early marker of lymphocyte activation, its role seems to be more complex, also involving immunoregulatory functions [52]. Importantly, decreased CD69 levels on lymphoma cells have previously been found in more Th2-skewed CTCL lesions [53], a phenotype associated with progressing disease [9].

The thioredoxin-interacting protein *TXNIP* can mediate oxidative stress, inhibit cell proliferation, induce apoptosis [54], and has also been described as an inhibitor of NK cell-mediated macrophage activation [55]. However, decreases of *TXNIP* in advancing lesions is consistent with its putative tumor suppressor function [56], as forced expression inhibits malignant proliferation in CTCL-derived cell lines [57]. Tristetraprolin/TTP (*ZFP36*), a TIS11 family member, promotes the degradation of several cytokines via binding to AU-rich elements in the 3′ untranslated regions of their mRNA. TTP is known to act as a tumor suppressor and key regulator of inflammatory responses [58, 59]. Decreased TTP levels have been demonstrated in malignancies with MYC involvement, and restoring its levels counteracted cancer development [60]. Thus, decreasing levels of TTP/*ZFP36* and *TXNIP* in malignant clones indicate loss of tumor-suppressive functions.

Keratinocyte-derived IL-7 has been described as a potent growth factor for CTCL [61–63], and regulation of its receptor IL7R has previously been associated with IL-2 cytokine signaling [64, 65]. The specific decrease in *IL7R* that we found in malignant clones, but not benign bystander cells, might therefore indicate a change in cytokine responsiveness over time. In line, malignant MF cells are considered more and more cytokine independent with advancing disease, and IL-7 might thus only impact on malignant cells in early, but not advanced disease stages [66].

The role for *HSPA1A,* coding for heat shock 70 kDA protein 1 (HSP72), is less obvious. HSP72 is involved in DNA repair and the guidance of protein folding as a chaperone [67], has previously been reported to be increased in aggressive versus non-aggressive MF [68], and to dampen T cell mediated inflammatory reactions in vitro [69]. However, this gene product is believed to have a dual role in cancer cells, as intracellular HSP72 protects malignant cells by interfering with apoptotic pathways, while membrane-associated and extracellular HSP72 can elicit antitumor immune responses [70]. Given these discrepancies, the exact role of this mediator in MF remains to be elucidated. Nevertheless, the fact that *CXCR4, CD69, HSPA1A, ZFP36, IL7R* and *TXNIP* were concomitantly decreased in TCR-αβ and TCR-γδ tumor cells of advanced MF lesions, and the observation that *CD69, HSPA1A* and *ZFP36* were increased in early-stage MF patients, suggests that this marker panel reflects a general mechanism of skin lesion progression in MF in a continuous fashion from longstanding indolent to more aggressive late-stage disease. However, the common denominator regulating these marker changes in MF cells remains to be elucidated. Much to our surprise, five out of seven MF patients harbored substantial numbers of malignant cells also in clinically uninvolved skin. In line with a potentially more “silenced” phenotype, these nonlesional tumor cells harbored elevated levels of *CXCR4, CD69, HSPA1A, ZFP36, IL7R* and *TXNIP* when compared to matched lesional skin. Whether there are differences in gene expression between areas of treated MF (i.e. “postlesional”) vs. locations that have never been clinically involved (“never-lesional” skin) needs to be determined in larger patient cohorts. Nevertheless, this finding sheds a new light on future curative CTCL treatment approaches, that will need to take into account a tumor cell burden well beyond merely visibly involved skin. Importantly, we found a population of *IGFL2*+ *KIR3DL2*+ tumor cells that was largely absent in nonlesional MF or healthy control skin, which might be crucially involved in the formation of actively inflamed, clinically visible MF lesions. In line, *IGFL2* has previously been described as a marker overexpressed in Sézary cells [32], and *KIR3DL2* [71] is currently being investigated in clinical trials as therapeutic target for CTCL, showing early promising results [72].

In contrast to the expanded clone, we did not find consistent transcriptomic regulation within the microenvironment across patients, and receptor/ligand pairs of lymphoma cells with benign cells were expressed in a very heterogeneous fashion. Yet, we found some limited downregulation of the *CXCL12-CXCR4* and *CCL5-CCR4* immune axes in advancing lesions, which might indicate decreasing interaction with fibroblasts and cytotoxic T cells, respectively. Interestingly, early-stage MF tumor cells showed a more cytotoxic phenotype when compared to advanced-stage MF patch lesions. In line, indolent lymphomas such as lymphomatoid papulosis have been described to preferentially express such molecules in contrast to more aggressive CTCL [73], suggesting a potential involvement in indolent lesion behavior. In line with previous publications, we found a shift towards type-2 inflammation in advancing lesions, as well as increasing numbers of B cells, which might be relevant players for lesion progression [9, 74]. We were surprised not to find significant regulation of checkpoint inhibitors or their ligands in our advanced-stage patch vs. plaque/tumor analyses, as PD1 gene mutations have been described to drive aggressive behavior in CTCL [75]. Nevertheless, we found *CTLA4* to be strongly upregulated in helper T cells of nonlesional MF, which might have a role in anti-inflammatory properties of clinically unaffected MF skin. We also found some residual inflammation in nonlesional MF vs. healthy control skin, which might also have a role in tumor cell control, a phenomenon that needs further elucidation in larger patient cohorts.

## Conclusions

Taken together, we identified a characteristic panel of markers associated with cutaneous disease progression in MF. Such potential drivers of disease might constitute ideal targets for future drug therapy with a new treatment strategy of preventing disease progression by preserving a more indolent cancer biology.

## Supplementary Information

**Additional file 1: Supplementary Methods**.

**Additional file 2: Table S1**.

**Additional file 3: Table S2**.

**Additional file 4: Table S3**.

**Additional file 5: Table S4**.

**Additional file 6: Table S5**.

**Additional file 7: Table S6**.

**Additional file 8: Table S7**.

**Additional file 9: Table S8**.

**Additional file 10: Table S9**.

**Additional file 11: Table S10**.

**Additional file 12: Table S11**.

**Additional file 13: Table S12**.

**Additional file 14: Table S13**.

**Additional file 15: Table S14**.

**Additional file 16: Table S15**.

**Additional file 17: Table S16**.

**Additional file 18: Table S17**.

**Additional file 19: Table S18**.

**Additional file 20: Table S19**.

**Additional file 21: Table S20**.

**Additional file 22: Table S21**.

**Additional file 23: Table S22**.

**Additional file 24: Table S23**.

## Data Availability

The 10X Genomics datasets generated during this study is publicly available via Gene Expression Omnibus GSE173205.

## Abbreviations

CTCL: Cutaneous T-cell lymphoma
MF: Mycosis fungoides
TCR: T-cell receptor
T_RM_: Tissue resident memory T cell
scRNA-seq: Single-cell RNA sequencing
TCRA: T-cell receptor alpha locus
TCRB: T-cell receptor beta locus
