# Supplementary material for: Single-cell RNA sequencing reveals markers of disease progression in primary cutaneous T-cell lymphoma
Source: Mol Cancer. 2021 Sep 28;20:124. doi: 10.1186/s12943-021-01419-2 (PMC8477535; doi:10.1186/s12943-021-01419-2)
Supplement: Supplementary file 1 — Additional file 1: Supplementary Methods. [file 12943_2021_1419_MOESM1_ESM.docx]

**SUPPLEMENTAL METHODS**

**scRNA-seq data analysis**

*Alignment, VDJ annotation and quality control*

10X Genomics Cell ranger (v3.0.2, RRID: SCR_017344) standard pipeline was used for primary data analysis. Sequences were demultiplexed with the command “mkfast” and reads from each transcriptome library were aligned to the human reference genome assembly “refdata-cellranger-GRCh38-3.0.0”. Output of this pipeline was a raw UMI (Unique molecular identifier) count matrix, including counts for all the droplets, and a filtered UMI count matrix, including only those droplets which are likely to contain at least one cell. Assembly of reads from both, αβ and γδ TCR receptor libraries were performed using the 'cellranger vdj' pipeline in the reference-assisted mode using the vdj_GRCh38_alts_ensembl-3.1.0 reference version, respectively. Output generated by this pipeline included annotations of the assembled contigs according to the reference and a table grouping cells into clonotypes. Clonotype tables, TCR amino acid sequences and respective filtered gene expression matrices were loaded into R version 3.6.3 (2020-02-29, RRID: SCR_001905) for further analyses. Quality control was done separately for each sample by removing cell doublets using R package scran (v1.14.6, RRID: SCR_016944) (1). The command “doubletCells” simulates thousands of doublets by adding together two randomly chosen single cell profiles. For each cell the number of simulated doublets in the neighborhood was recorded and used as input to calculate a doublet score. Threshold to filter putative doublets was set to three times the median absolute deviation of the doublet score and all cells with a higher score were discarded. Seurat package (version 3.1.4, RRID: SCR_016341) (2, 3) was used for additional QC steps and merging/integration of samples. Data from TCR receptor sequencing and transcriptome sequencing were merged by adding clonotype frequency and CDR3 amino acid sequences to the metadata column of the Seurat objects. Additional filtering steps were carried out discarding cells with high mitochondrial content and very low or very high numbers of expressed genes.

*Integration, visualization and clustering*

Biopsy samples were ‘sctransformed’ and integrated with the Seurat SCT integration workflow using 3000 variable genes as integration features as described by Hafemeister & Satija (4). Following integration, principal component (PC) analysis was done and PCs were selected based on the explained variance by each principal component (elbow plot) as input for dimension reduction and clustering using the Louvain algorithm. For integrated patch and plaque/tumor samples from MF309, MF311, and MF312 the first 22 PCs and a resolution of 1 were used. For separate integrations of MF309 (patch, tumor and follow-up), MF311 (patch, plaque and erythroderma), MF312 (patch, plaque and treated lesion), and MF γδ (patch, plaque), the first 20, 20, 23 and 20 PCs, and a resolution of 0.8, 1, 0.8 and 0.8 were used, respectively. Integration of three samples from advanced-stage patch lesions (MF309, MF311, MF312) with three samples from early-stage disease was done using 22 PCs and a resolution of 0.7, and for the integration of seven lesional and nonlesional MF samples 30 PCs and a resolution of 0.5 was used.

Visualization of clusters in two-dimensional space was achieved by Uniform Manifold Approximation and Projection (UMAP) and we annotated the clusters with the corresponding cell types with the “FindAllMarkers” command (Wilcoxon Rank Sum Test) and looking at top expressed genes of each cluster. Differential gene expression was calculated using the FindMarkers command with logistic regression as test and percentage of mitochondrial genes as latent variable. P-values were adjusted for multiple comparisons with Bonferroni correction. Volcano plots were generated using the EnhancedVolcano R package (version 1.6.0, RRID:SCR_018931) (5). Bar plots and correlation plots were generated using ggplot (package ggplot2, version 3.2.1, RRID:SCR_014601); all other plots were generated using the Seurat package.

Lymphocytes were subsetted according to cluster annotations (all clusters containing T cells), and actual cell types were further subsetted according to marker expression and T cell receptor sequence in cytotoxic T cells (polyclonal T cell receptors, *CD8A+, FOXP3-)*, benign helper T cells (polyclonal T cell receptors, *CD4+, FOXP3-),* regulatory T cells (polyclonal T cell receptors, *FOXP3+*), malignant T cells (top monoclonal T cell receptors). In the comparison between lesional and nonlesional MF samples, lymphocyte clusters were newly subclustered (runPCA on subset) with 12 PCs and a resolution of 0.5. The same strategy was used in the comparison between early and advanced lesions with 20 PCs and a resolution of 0.4 to find new clusters. Resulting clusters were again annotated according to top upregulated markers (FindAllMarker as mentioned above) and TCR receptor abundance.

**Inference of cell-cell interactions from scRNA-seq data**

To gauge putative interactions between malignant cells and cells of the microenvironment we made use of co-expression between receptor-ligand pairs in the malignant clusters (TC-2, TC-3, TC-4, TC-6, TC-7, TC-8, TC-9) and other cell clusters present in the same sample. Dendritic cells DC-1, DC-2, DC-3, keratinocytes KC-1 and KC-2, fibroblasts FB-1 to FB-5, and malignant T cell clusters were pooled for analyses. To this end, we obtained ligand-receptor pairs (*R*) from the CellPhoneDB database (RRID: SCR_017054, data obtained from https://github.com/Teichlab/cellphonedb, commit 05d95adc6ebb3546f302c8ec422d1b86594cd8df) and examined the expression of each pair in each combination of cell types, similar to what has been described before (6). Briefly, we first calculated the mean expression level (scaled log counts, from Seurat) of each receptor or ligand per group of cells. To reduce potential confounding effects of different cell numbers in each sample, we took 100 random samples of 1,000 cells each for this analysis. For each random sample, we furthermore generated 100 samples with randomly permutated cluster labels as controls (100*100 = 10,000 total; these were used to calculate empirical *p*-values later). As in CellPhoneDB, we set the mean expression value to 0 if only a small fraction of cells (<10%) in the cluster expressed the gene. We then calculated the interaction score *I_R_(X,Y)* for each receptor-ligand pair *R* between each combination of cell clusters *X* and *Y* as the mean of the mean expression levels if both where greater than 0. If one mean expression level was 0, also the interaction was set to 0. We then used the interaction scores in real and permutated samples to estimate empirical *p*-values as the proportion of random samples that had an *I_R_(X,Y)* greater or equal to the score of the real sample. For the analysis presented in the paper, we focused on significant interactions (FDR-adjusted p-value<= 0.05) between malignant T cells and other cell types. All analyses were performed using custom code in R (v4.0.3). Network diagrams and heatmaps were generated using the *igraph* (v1.2.6, RRID: SCR_019225) (8(7) and *pheatmap* (v1.0.12, RRID: SCR_016418) packages in R, respectively.

**Immunofluorescence (IF) microscopy**

Human skin punch biopsies were embedded in optimal cutting temperature (O.C.T.) compound (Tissue-Plus, Scigen Scientific, Gardena, CA), and stored at -80°C until further processing, as previously described (8). Samples were cut into 5 µm sections and mounted on SuperFrost Plus adhesion slides (Thermo Scientific, Waltham, MA), air dried for 30 minutes, and fixed in ice-cold acetone (Sigma-Aldrich, St. Louis, MO) for 10 minutes. To minimize background, sections were incubated with PBS containing 2% BSA (Sigma-Aldrich) and 2% goat serum (Dako, Glostrup, Denmark) for 20 minutes in a humid chamber at room temperature (RT). The following primary antibodies were used: Anti-CD68 (5 µg/ml, clone KP1, Dako), anti-CD11c (1:10, Bu15, Biolegend), anti-TCR Vβ21.3 (1:10, Beckman Coulter), anti-CXCR4 (1:10, 12G5, Biolegend), anti-CD127 (1:10, A019D5, Biolegend), anti-CD69 (1:10, FN50, Biolegend), anti-CD3 (1:10, SK7, BD Biosciences). The following secondary antibodies were used, if needed: Alexa Fluor (AF) 546-conjugated goat-anti-mouse IgG (1:400, Thermo Fisher Scientific); AF488-conjugated anti-fluorescein/Oregon Green (1:400, Thermo Fisher Scientific). Antibodies were incubated at room temperature for 30 minutes, and counterstained for 5 min with 1 µg/ml 4,6-diamidino-2-phenylindole dihydrochloride (DAPI) (Roche Diagnostics, Mannheim, Germany), and mounted in aqueous mounting medium PermaFluor (Thermo Fisher Scientific).

Skin sections were scanned at room temperature using a TissueFAXS v6 imaging system (TissueGnostics GmbH, Vienna, Austria) with a Zeiss Axio Observer Z1 microscope (Carl Zeiss Inc, Jena, Germany), Zeiss Plan-Neofluar objectives (primary objective 20x/0.5, ocular objective 10x), Spectra III 8-LCR-XN light engine and a PCO PixelFly monochrome camera (PCO, Kelheim, Germany) and Pixelink PL-623 color camera (Pixelink, Rochester, NY). The scanning was conducted in fluorescence mode using the filters detecting DAPI, green fluorescent protein (GFP), cyanine (Cy) 3 and Cy5 fluorochromes. Scan settings were adapted to the respective isotype controls. Images were exported from the TissueFAXS v6 software as TIFF.

**Isolation of T cells for cell sorting and subsequent quantitative RT-PCR**

Four-millimeter skin punch biopsies were minced and digested for 30min using 500 U/ml Collagenase IV (Worthington Biochemical, Lakewood, NJ) and 50 U/ml DNAse I (Sigma Aldrich) in RPMI 1640 medium (Gibco, Thermo Fisher Scientific, Waltham, MA) at 37°C. The cell suspension was sieved using a 70µM cell strainer (Corning, Corning, NY) and stained using the following antibodies: Anti-CD45 PE-CF594 (clone HI30, BD Biosciences), anti-CD7 APC (clone CD7-6B7, Biolegend), anti-CD4 BV605 (clone SK3, Biolegend), anti-CD3 BV711 (clone SK7, Biolegend), anti-CD8 BV785 (clone SK1, Biolegend), anti.FCR Vβ21.3 FITC, including appropriate isotype controls. For live/dead discrimination cells were counterstained with 7-Aminoactinomycin (7-AAD, EMD Millipore Corp., Merck) at a final concentration of 5µg/ml. The cell suspensions were analyzed on a BD FACS Aria III flow cytometer (BD Biosciences, Franklin Lakes, NJ) and cell populations showing a malignant phenotype, according to individual marker losses such as CD4/CD8 negativity or CD7 loss (Table 1) and/or specific TCRB-antibodies, were sorted directly into TRIzol (Sigma Aldrich) reagent for RNA isolation. RNA was harvested according to the manufacturer’s instructions, and was reverse transcribed using the First Strand cDNA Synthesis Kit for RT-PCR (AMV) (Roche, Basel, Switzerland‎) according to the manufacturer’s instructions. Generated cDNA was then subjected to quantitative Real-Time PCR using TaqMan Gene Expression Assays B2M: Hs00187842_m1, CD69: Hs00934033_m1, CXCR4: Hs00976734_m1, HSPA1A: Hs00359163_s1, ZFP36: Hs00185658_m1, IL7R: Hs00902334_m1, and TXNIP: Hs00197750_m1 (Thermo Fisher Scientific) on a StepOnePlus Real-Time PCR System (Applied Biosystems, Foster City, CA). The samples were quantified by the StepOne Software v. 2.3 (Applied Biosystems) and calculated using B2M as a reference gene.

**SUPPLEMENTARY FIGURES**


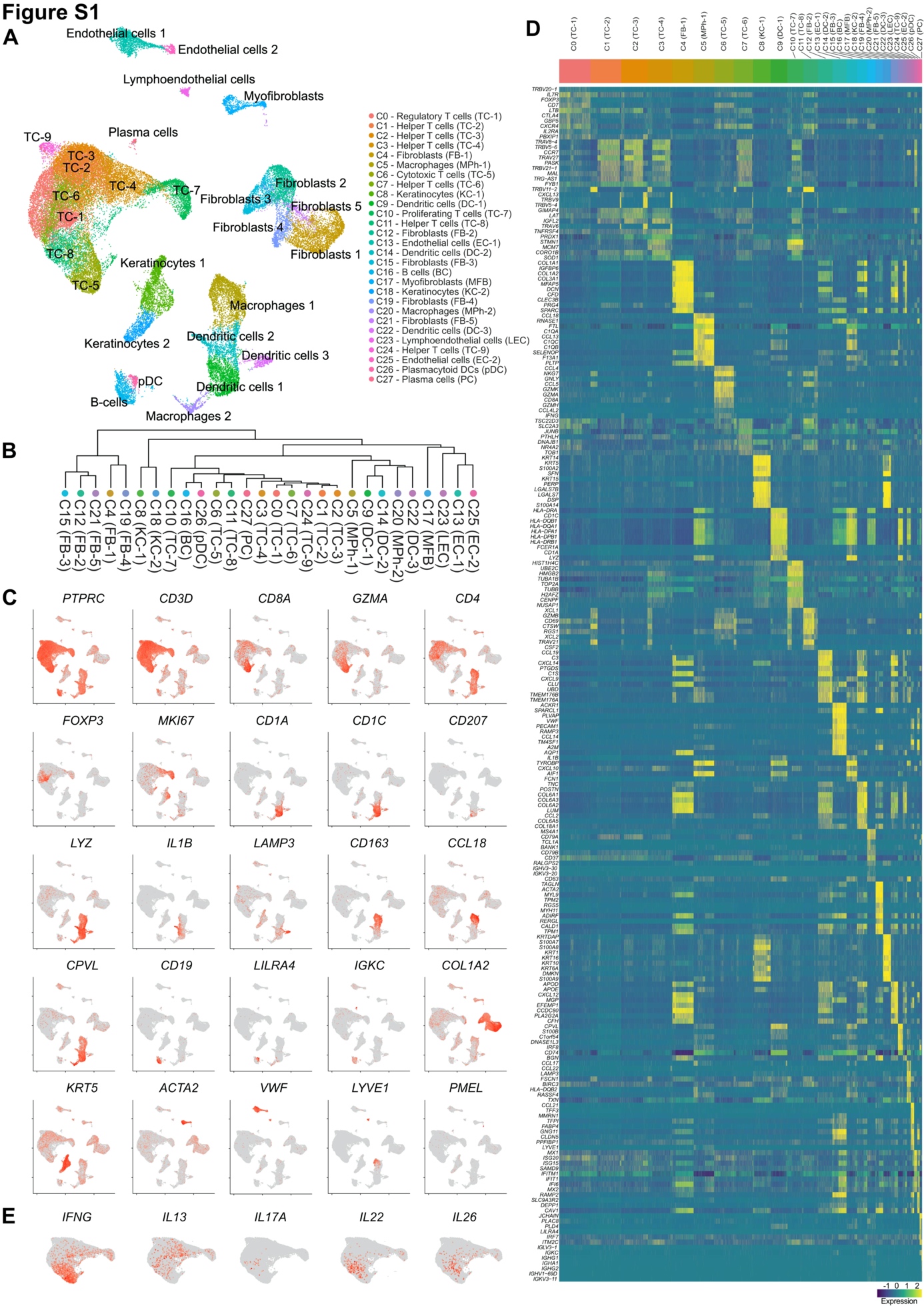


**Figure S1. Overview of skin cell populations as defined by marker expression, related to Figure 1, Tables S1 and S2. (A)** UMAP of 47,172 cells integrated from 3 patch and 3 plaque/tumor skin biopsies of three different patients MF309 (9,577), MF311 (26,921), and MF312 (10,674) according to similarity of their transcriptome, resulting in 28 different color-coded clusters (C0-C27). **(B)** Unsupervised hierarchical clustering showing relatedness of cell clusters (average gene signatures; correlation distance metric and average linkage). **(C)** Combined feature plots showing expression distribution for canonical marker genes. Intensity of normalized expression levels for each cell is color-coded (red) and overlaid onto UMAP plots. **(D)** Heat map displaying the top 10 differentially expressed genes (according to smallest adjusted p-value and average log fold change with Bonferroni correction for each cluster compared with the rest of the dataset); upregulation is indicated in yellow, and downregulation in blue/green; gene names are shown on the left, cluster names on top. **(E)** T cell clusters depicting cytokines typically associated with type 1 (*IFNG*), type 2 (*IL13*), type 17 (*IL17A, IL26*) and type 22 (*IL22*) immune responses.


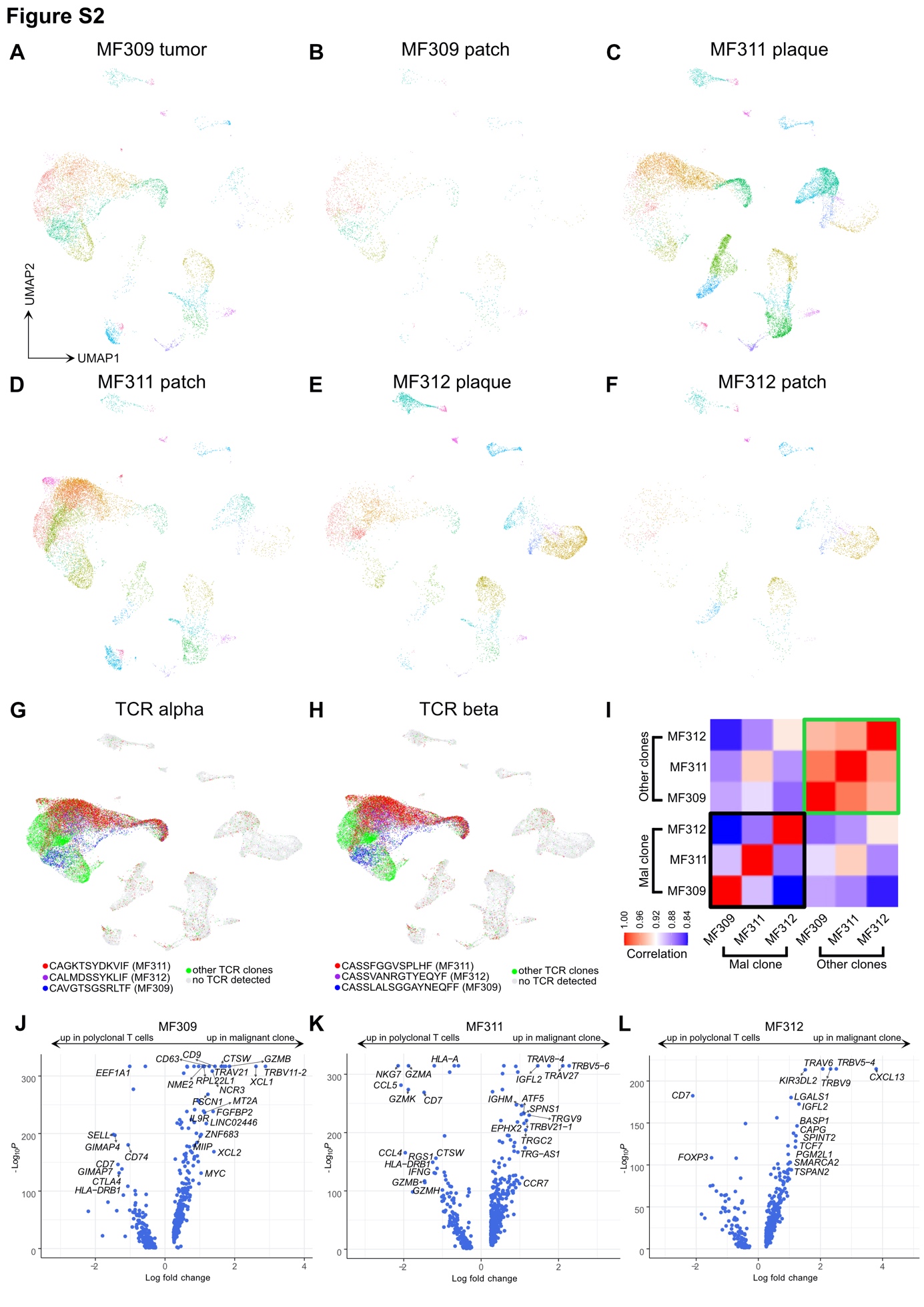


**Figure S2. Sequences and gene expression of malignant clones and polyclonal cells, related to Figure 2, Tables S1 and S2.** **(A-F)** UMAP plots of integrated MF samples from patch and plaque/tumor lesions of patients MF309, MF311, and MF312, split by sample and color coded by cell clusters. **(G-H)** UMAP plots of integrated MF samples; cells with the top expanded T-cell receptor α or β chain CDR3s amino acid sequence per patient are colored in blue (MF309), red (MF311), and purple (MF312); the remaining polyclonal α or β TCRs are labelled in green, and cells without detectable TCR are displayed in grey. **(I)** Correlation heat map using Pearson’s correlation for transcriptomic properties (average gene expression) of mono- and polyclonal T cells of each patient, demonstrating close correlation between polyclonal (green box), but less so in monoclonal populations (black box) among patients. Color intensity reflects Pearson’s correlation coefficient. Mal clone: Malignant clone. **(J-L)** Volcano plots of differentially expressed genes between the malignant clone vs. polyclonal T cells with a detectable TCR, as calculated by logistic regression with Bonferroni correction, and depicted as log fold change (FCH).


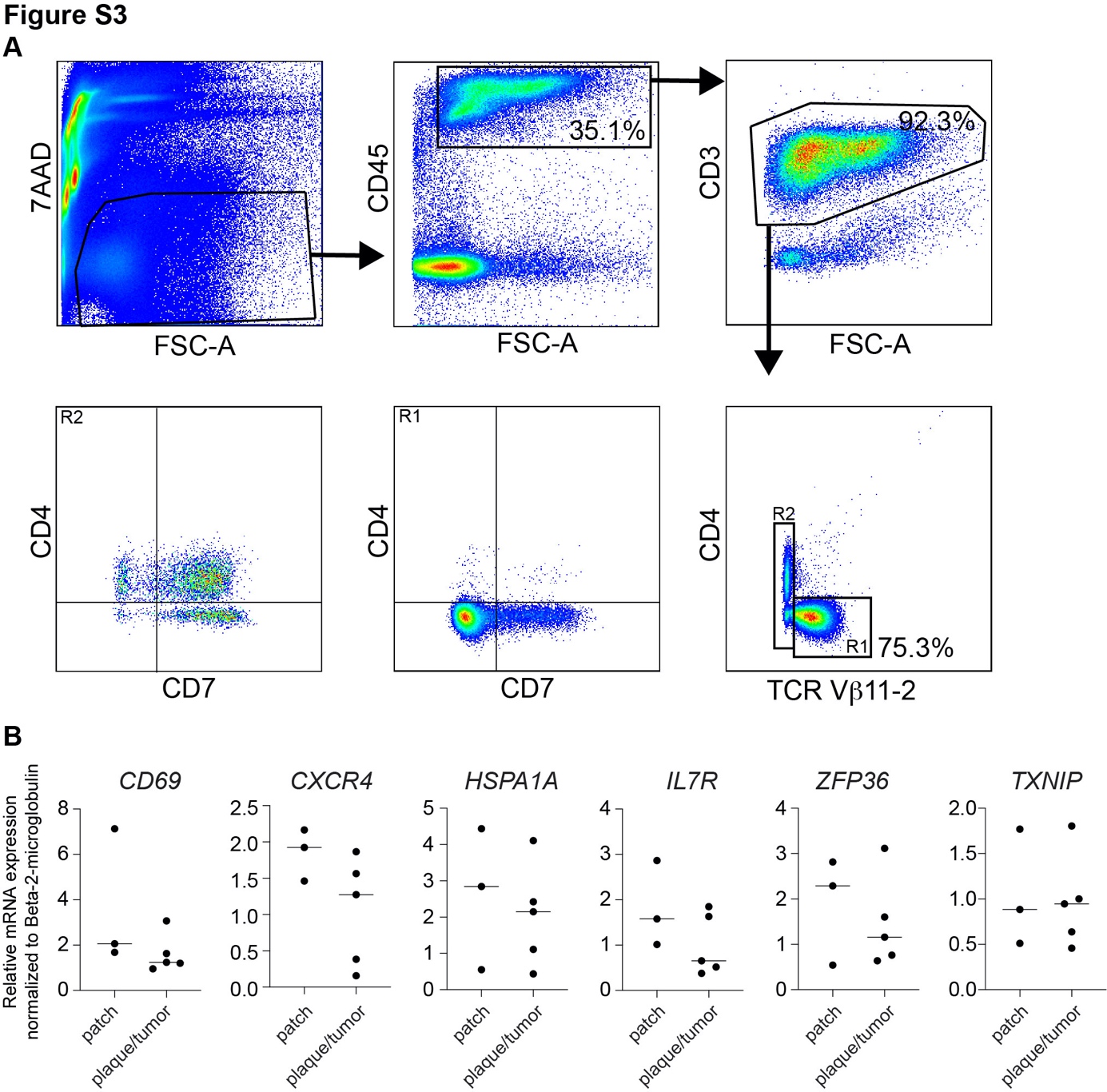


**Figure S3. Isolation and quantitative RT-PCR analysis of skin tumor cells. (A)** Gating strategy for the isolation of malignant T cells, exemplified for the case of MF309 (gate R1). Specific isolation protocols were derived from individual malignant cell phenotypes as listed in Table 1 by using specific TCRs and/or marker characteristics (e.g. loss of CD4, CD8, CD7), as needed for each patient. **(B)** Quantitative RT-PCR from isolated MF lymphoma cells from patch and plaque/tumor lesions; mRNA levels normalized to beta-2-microglobulin (B2M); horizontal line denotes median; each dot represents one single patient sample (patch: MF309, MF311, P182; plaque/tumor: MF309, MF311, P15, P107, P182).


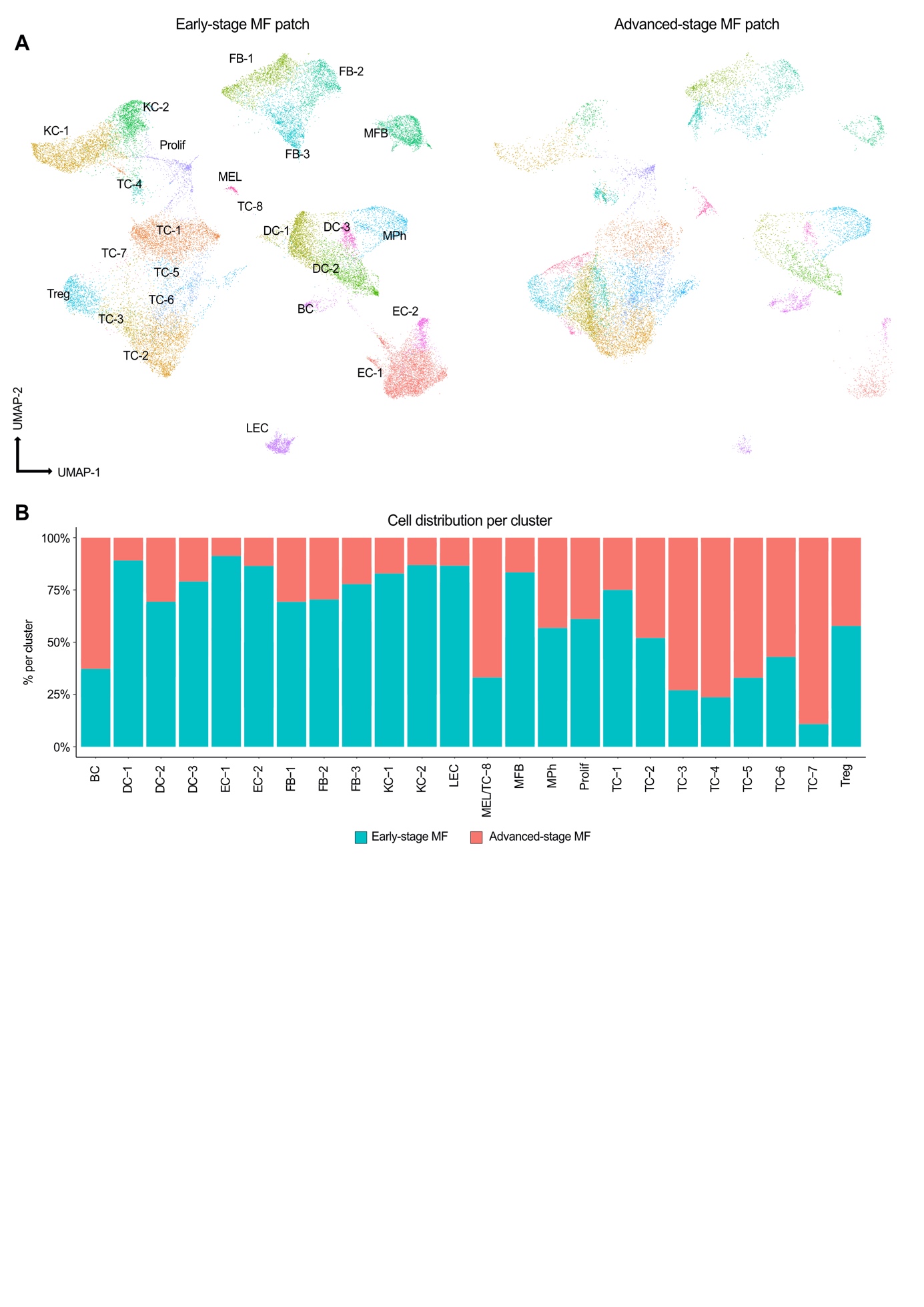


**Figure S4 Comparison of patch lesions from advanced-stage vs. early-stage MF.** **(A)** UMAP of 51,387 cells integrated from three early-stage and three advanced-stage MF patches according to similarity of their transcriptome, resulting in 24 different color-coded clusters and split according to tissue of origin. **(B)** Bar plot showing relative distribution of cells within individual clusters. TC T cells; Treg regulatory T cells; BC B cells; KC Keratinocytes; FB Fibroblasts; DC Dendritic cells; MPh Macrophages; MFB Myofibroblasts; EC Endothelial cells; LEC Lymphoendothelial cells; Prolif Proliferating cells; MEL Melanocytes; UMAP: Uniform Manifold Approximation and Projection.


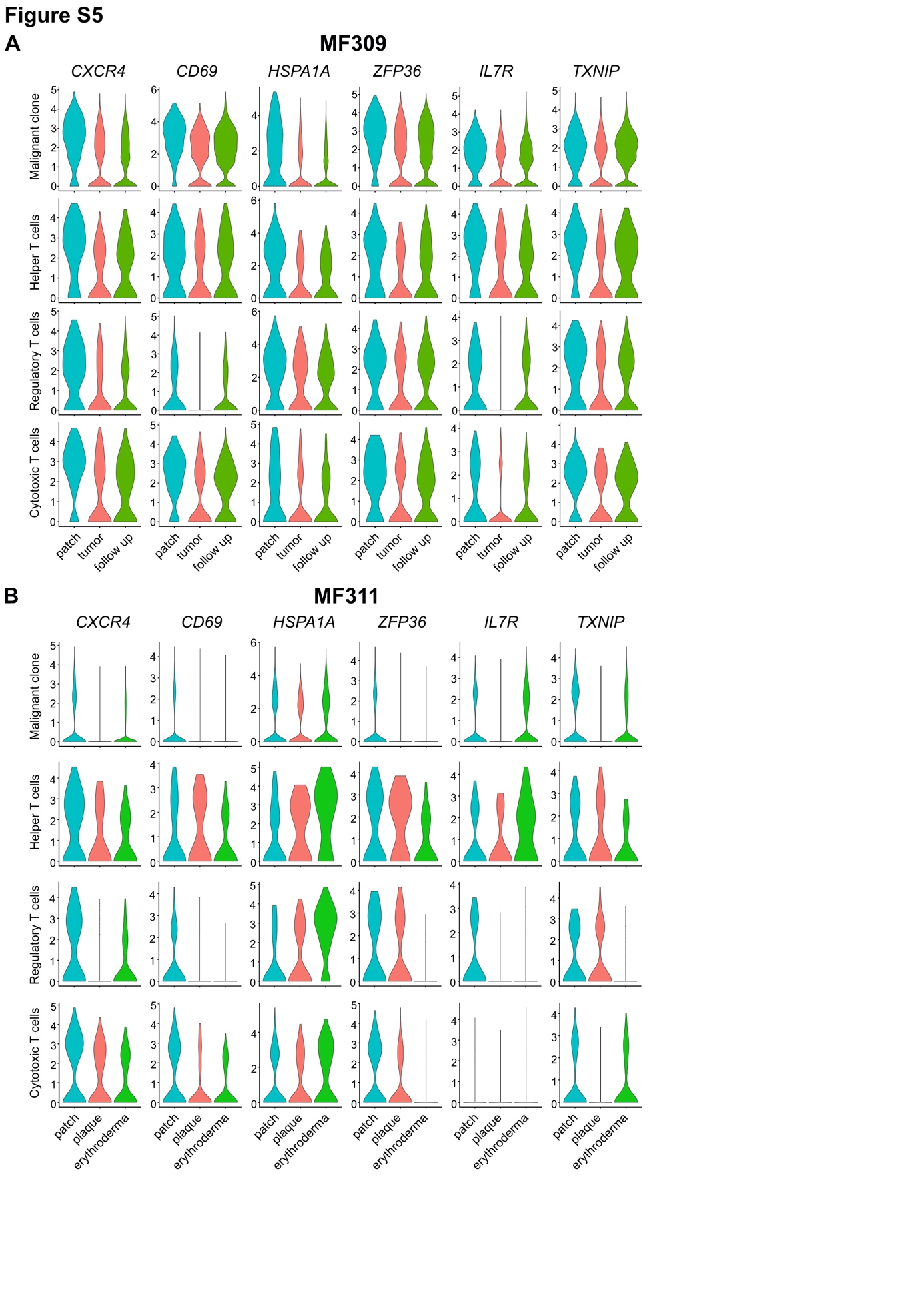


**Figure S5.** **Gene expression of *CXCR4, CD69, HSPA1A, ZFP36, IL7R* and *TXNIP* in T cell sub-populations,** **related to Figure 7.** Violin plots of T cell sub-populations from **(A)** patient MF309 and **(B)** patient MF311, showing distribution of normalized gene expression levels of the six marker genes in patch (turquois), plaque/tumor (red), and follow-up (green) lesions. Malignant clone: top expanded clone. Helper T cells: *CD4+ FOXP3-* cells with polyclonal TCRs. Regulatory T cells: *FOXP3+* cells with polyclonal TCRs. Cytotoxic T cells: *CD8+ FOXP3-* cells with polyclonal TCRs.


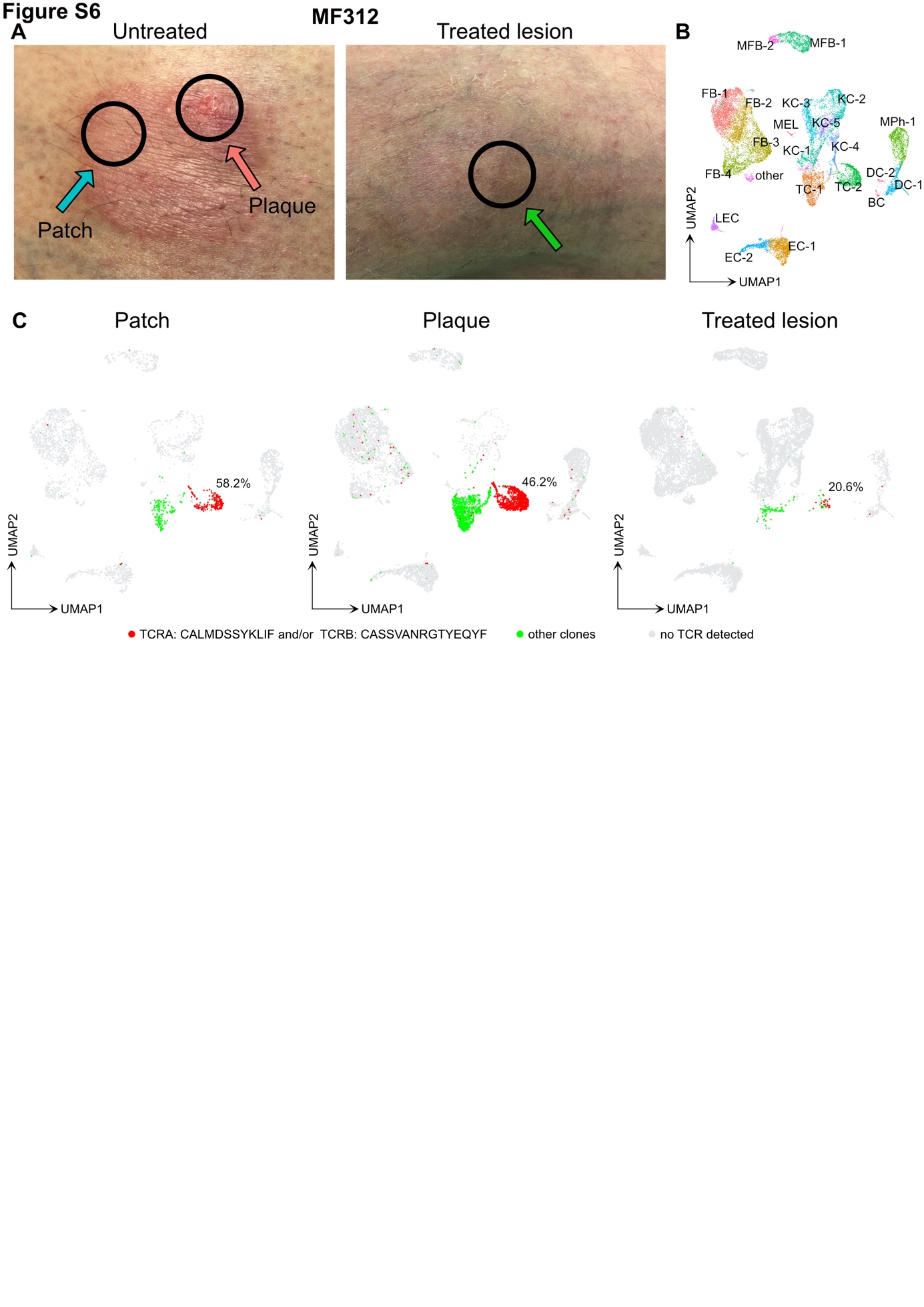


**Figure S6. Characterization of post-lesional skin after successful topical treatment in patient MF 312. (A)** Pictures of MF lesions from patient MF312 before and after topical treatment with chlormethine gel. Black circles indicate the location the biopsy was taken from. **(B)** UMAP of 20,416 cells integrated from three MF312 skin biopsies according to similarity of their transcriptome, resulting in 22 different color-coded clusters. **(C)** UMAP plots of samples colored according to most common monoclonal TCR (red), polyclonal αβ TCRs (green) and cells without detectable TCR (grey). Percentages denote frequencies of malignant cells among all TCR+ cells for each plot. TC T cells; BC B cells; KC Keratinocytes; FB Fibroblasts; DC Dendritic cells; MPh Macrophages; MFB Myofibroblasts; EC Endothelial cells; LEC Lymphoendothelial cells; pDC Plasmacytoid dendritic cells; MEL Melanocytes.

**
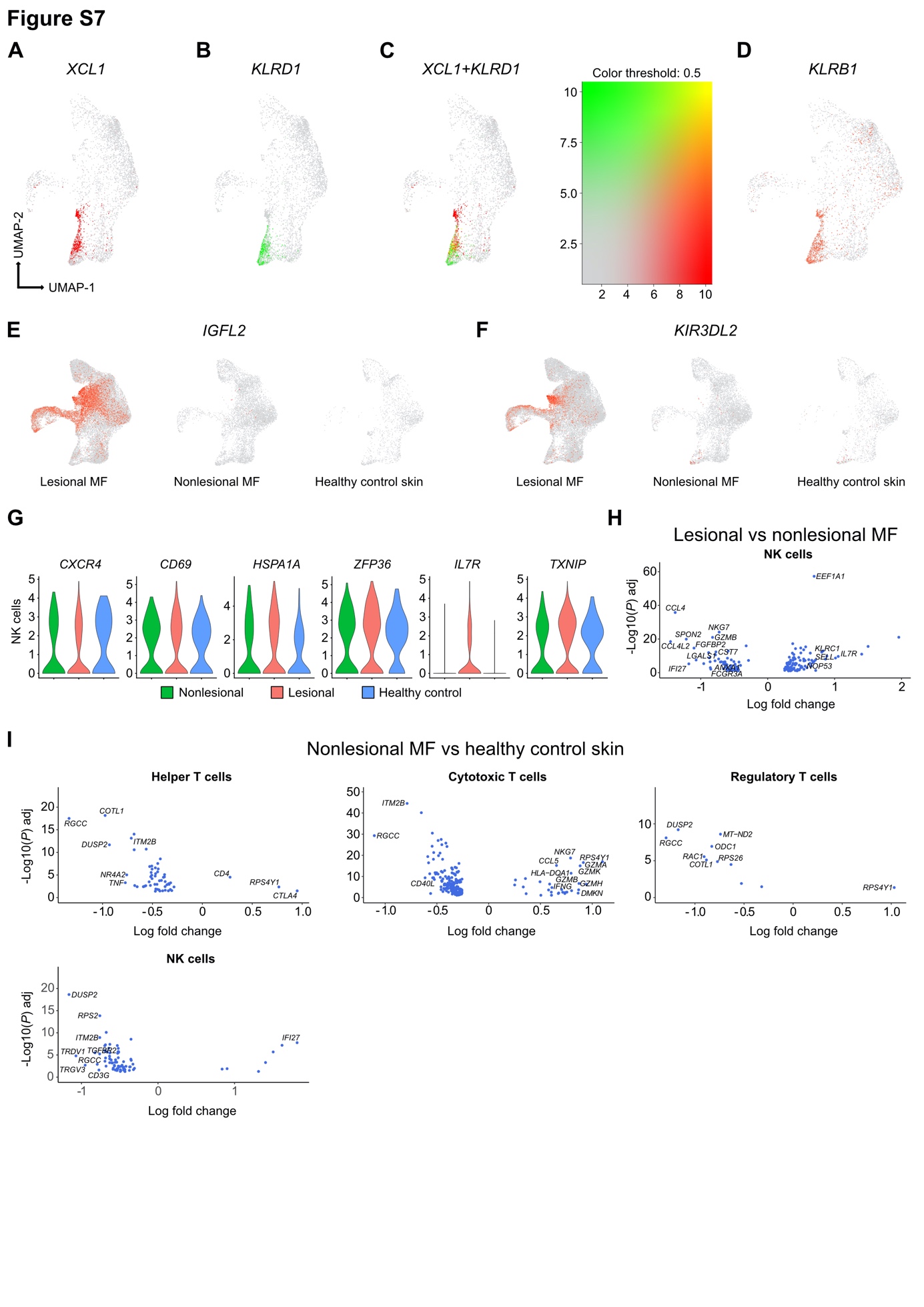
**

**Figure S7. Comparison of lesional with nonlesional MF and healthy control skin. (A-C)** Combined feature plots of (A) *XCL1* and (B) *KLRD1* overlaid onto TCR- CD3D-cells from Figure 8A; (C) NK cells are visible as *KLRD1+* cells in green, or *XCL1+ KLRD1+* cells in yellow; other innate lymphoid cells appear as *KLRD1- XCL1+* cells in red in the overlay plot. **(D)** Feature plot depicting *KLRB1+* cells overlaid onto TCR- CD3D- cells from Figure 8A. **(E-F)** Combined feature plots showing expression of *IGFL2* and *KIR3DL2* in T cell clusters. Normalized expression level for each cell is color-coded (red) and overlaid onto UMAP plots of cells from Figure 8A. **(G)** Violin plots showing distribution of normalized gene expression levels in nonlesional MF (green), lesional MF (red) and healthy control skin (blue) for NK cells. **(H)** Volcano plot showing differentially expressed genes (DEGs) of NK cells between lesional and nonlesional MF biopsies. **(I)** Volcano plot showing differentially expressed genes (DEGs) of polyclonal helper, cytotoxic and regulatory T cells, as well as NK cells, between nonlesional MF and healthy control biopsies. Differential gene expression was defined as log fold change >⏐0.25⏐ and an adjusted p<0.05 as calculated by logistic regression and Bonferroni correction. Regulatory T cells: *FOXP3+* cells with polyclonal TCRs. Cytotoxic T cells: *CD8A+ FOXP3-* cells with polyclonal TCRs. Helper T cells: *CD4+ FOXP3-* cells with polyclonal TCRs. NK cells: TCR negative cells within cluster “NK” of UMAP plots from Figure 8A. UMAP: Uniform Manifold Approximation and Projection.

**
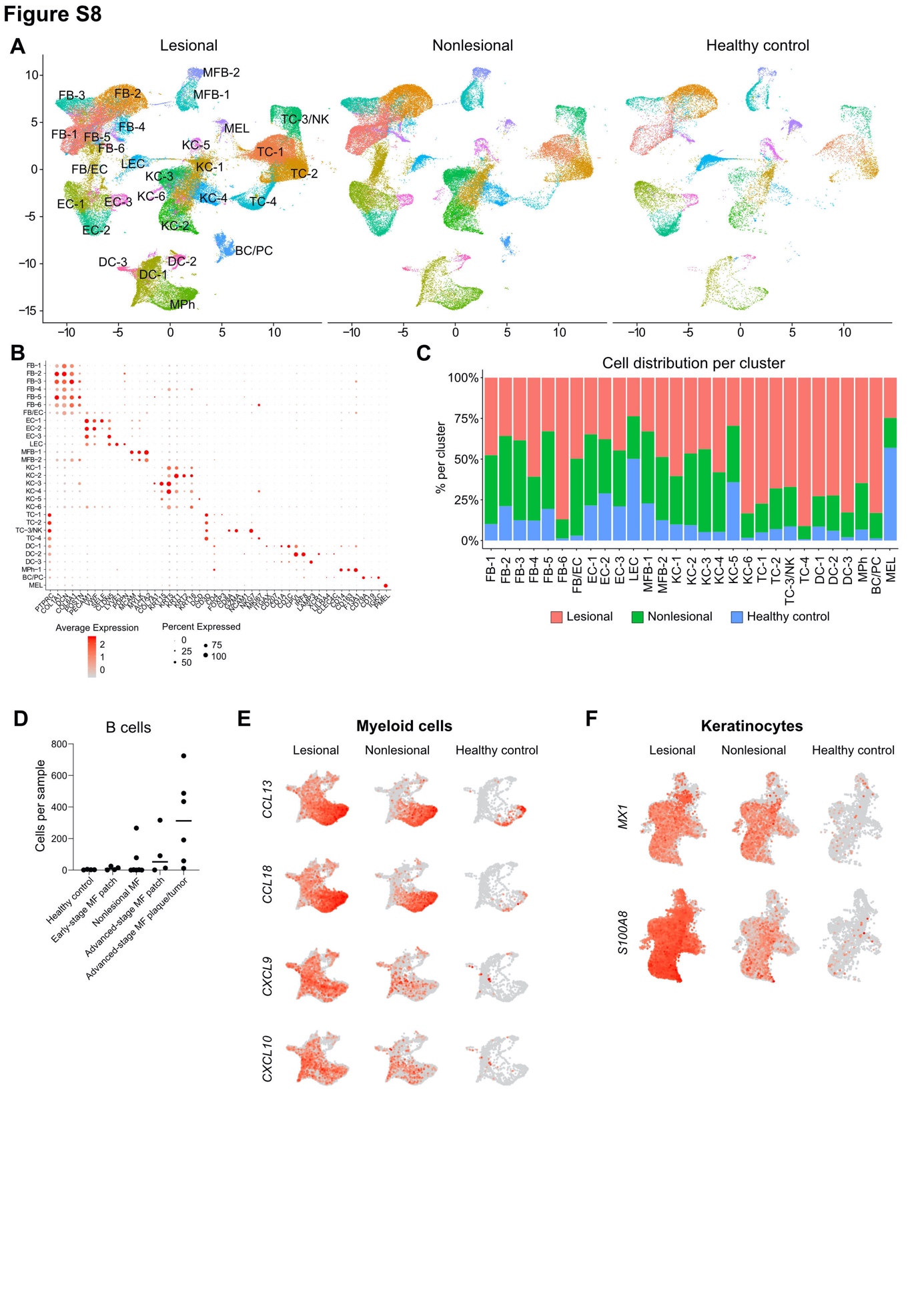
**

**Figure S8. Comparison of lesional with nonlesional MF skin biopsies as well as healthy control biopsies. (A)** UMAP of 150,584 cells integrated from seven patients with matched lesional and nonlesional MF, as well as four healthy control individuals, according to similarity of their transcriptome, resulting in 29 different color-coded clusters and split according to tissue of origin. **(B)** Dot plot of all cell clusters displaying average gene expression (red color) and frequency (circle size) of canonical markers for individual cell populations. **(C)** Bar plot showing relative distribution of cells within individual clusters. **(D)** Numbers of B cells as estimated across samples; cells were selected for TCR negativity, and defined by expression of *MS4A1* and/or *CD19* and/or *CD79A*. One dot represents one individual patient sample. **(E-F)** Combined feature plots showing expression of inflammatory markers in myeloid and keratinocyte clusters. Normalized expression level for each cell is color-coded (red) and overlaid onto UMAP plots. TC T cells; Treg regulatory T cells; BC B cells; KC Keratinocytes; FB Fibroblasts; DC Dendritic cells; MPh Macrophages; MFB Myofibroblasts; EC Endothelial cells; LEC Lymphoendothelial cells; Prolif Proliferating cells; MEL Melanocytes; UMAP: Uniform Manifold Approximation and Projection; MF Mycosis fungoides.

**SUPPLEMENTARY TABLE LEGENDS (For supplementary tables see separate excel files)**

**Table S1.** Cell numbers per cluster after QC filtering of respective samples that were used for patch vs plaque/tumor analyses.

**Table S2.** **Clustermarkers of plaque/tumor and patch lesions from patients MF309, MF311 and MF312, related to Figure 1 and Figure S1.** Top 10 differentially expressed genes according to highest log fold change ordered by smallest adjusted p-value using Wilcoxon Rank Sum Test with Bonferroni correction for each cluster, as compared to the rest of the dataset in all sequenced cells, integrated from patch and plaque/tumor samples of MF309, MF311 and MF312.

**Table S3.** **Comparison of tumor/plaque vs patch lesions in patients MF309, MF311 and MF312, related to Figure 3.** Differentially expressed genes between tumor/plaque vs patch lesions in malignant cells (“Mal clone” as defined by expressing the single top expanded TCR), regulatory T cells (TREG, *FOXP3+*), cytotoxic T Cells (Tc, *CD8A+ FOXP3-*) and T helper cells (Th, *CD4+, FOXP3-*) for each patient as defined by a logFC>|0.3| and adjusted p<0.05 using logistic regression with Bonferroni correction. **Summary** of differential gene expression analysis of 6 genes mutually downregulated in malignant clones of all three patients (plaque/tumor vs patch lesions), compared with respective benign T cell subsets for each individual.

**Table S4. Comparison of plaque/tumor vs patch lesions for each cluster, related to Figure 4A.** Integrated samples of MF309, MF311 and MF312 comparing tumor/plaque vs. patch lesions. Differential gene expression calculated using logistic regression and Bonferroni correction, defined as logFC>|0.25| and adjusted p-value<0.05.

**Table S5. Analysis of putative interactions between the malignant clone and non-malignant cells, as inferred by co-expression of ligand-receptor pairs (*R*) from CellPhoneDB, related to Figure 4D-E.** Cell-cell interaction scores for each indicated receptor-ligand and cell type pair in each sample, as well as differences between interaction scores in plaque/tumor vs. patch lesions, for patients MF309, MF311 and MF312. Interaction scores (*I_R_(X,Y)*) were inferred by co-expression of ligand-receptor pairs (*R*) from CellPhoneDB between malignant T cells (*X*) and each cell cluster (*Y*) in the same sample. Dendritic cells DC-1, DC-2, DC-3, keratinocytes KC-1 and KC-2, fibroblasts FB-1 to FB-5, and malignant T cell clusters were pooled for analyses. Only receptor-ligand pairs with a significant interaction score in at least one sample are shown (FDR-adjusted empirical p-value<= 0.05).

**Table S6.** **Characterization of gamma/delta MF lesions from patient MF318, related to Figure 5.** Cell numbers per cluster after QC filtering of patch and plaque lesions. **Clustermarker:** Top 10 differentially expressed genes according to highest log fold change ordered by smallest adjusted p-value using Wilcoxon Rank Sum Test with Bonferroni correction for each cluster, as compared to the rest of the dataset in all cells for the gamma/delta MF patient MF318. **DEG:** Differentially expressed genes between plaque and patch lesions in patient MF318 as calculated for malignant cells (as defined by expressing the single top expanded TCR), regulatory T cells (TREG, *FOXP3+*), cytotoxic T Cells (Tc, *CD8A+ FOXP3-*) and T helper cells (Th, *CD4+, FOXP3-*) as defined by a logFC>|0.25| and adjusted p-value<0.05 using logistic regression with Bonferroni correction. **Summary** of differential gene expression analysis (plaque vs. patch) of 6 selected genes downregulated in the malignant clone, compared to respective benign T cell subsets of MF318.

**Table S7.** **Differential gene expression between plaque and patch lesions in patient MF318 for each cluster, related to Figure 5F.** Comparison of plaque vs. patch lesions in the gamma/delta TCR MF patient MF318 for each cell cluster; differential gene expression analysis of scRNA-seq analyses as defined by logFC>|0.25| and adjusted p-value<0.05, using logistic regression and Bonferroni correction.

**Table S8. Comparison of T cells from patches of advanced-stage MF vs early-stage MF.** Cell numbers per cluster after QC filtering, corresponding to Figure 6B. **Clustermarker:** Top 10 differentially expressed genes according to highest log fold change ordered by smallest adjusted p-value using Wilcoxon Rank Sum Test with Bonferroni correction for each cluster, as compared to the rest of the dataset. **Differentially expressed genes** between advanced-stage MF vs early-stage MF patches, as calculated for malignant cells (as defined by expressing the single top expanded TCR), regulatory T cells (TREG, *FOXP3+*), cytotoxic T Cells (Tc, *CD8A+ FOXP3-*) and T helper cells (Th, *CD4+, FOXP3-*) as defined by a logFC>|0.25| and adjusted p-value<0.05 using logistic regression with Bonferroni correction. **Summary** of differential gene expression analysis of 6 selected marker genes, compared with respective benign T cell subsets.

**Table S9. Comparison of all cells from patches of advanced-stage MF vs. early-stage MF.** Cell numbers per cluster after QC filtering, corresponding to Figure S4. **Clustermarker:** Top 10 differentially expressed genes according to highest log fold change ordered by smallest adjusted p-value using Wilcoxon Rank Sum Test with Bonferroni correction for each cluster, as compared to the rest of the dataset. **Differentially expressed genes** between advanced-stage MF vs early-stage MF patches, as calculated for each cluster.

**Table S10.** **Follow-up characterization in patient MF309, related to Figure 7A-B.** Top 10 differentially expressed genes according to highest log fold change ordered by smallest adjusted p-value using Wilcoxon Rank Sum Test with Bonferroni correction for each cluster, as compared to the rest of the dataset in all cells for patient MF309 (integrated dataset of patch, tumor and follow-up lesion). **Mal clone follow-up vs patch:** Differentially expressed genes between follow-up sample and patch lesion in malignant cells of patient MF309 as defined by logFC>|0.25| and adjusted p-value<0.05 using logistic regression with Bonferroni correction. **Mal clone follow-up vs tumor:** Differentially expressed genes between follow-up sample and tumor lesion in malignant cells of patient MF309 as defined by logFC>|0.25| and adjusted p-value<0.05 using logistic regression with Bonferroni correction.

**Table S11. Comparison of follow-up (ulcerated tumor) vs patch lesion in patient MF309 for each cluster**, **related to Figure 7**. Differential gene expression analysis within cell types of scRNA-seq analyses as defined by logFC>|0.25| and adjusted p-value<0.05, using logistic regression and Bonferroni correction.

**Table S12.** **Comparison of follow-up (ulcerated tumor) vs initial tumor lesion in patient MF309 for each cluster, related to Figure 7B**. Differential gene expression analysis within cell types of scRNA-seq analyses as defined by logFC>|0.25| and adjusted p-value<0.05, using logistic regression and Bonferroni correction.

**Table S13.** **Follow-up characterization in patient MF311, related to Figure 7E-G.** Top 10 differentially expressed genes according to highest log fold change ordered by smallest adjusted p-value using Wilcoxon Rank Sum Test with Bonferroni correction for each cluster, as compared to the rest of the dataset in all cells for patient MF311 (integrated dataset of patch, plaque and erythroderma lesion). **TRA_CAGvsCAV**: Differentially expressed genes between TCRA1 (CAGKTSYDKVIF) vs TCRA2 (CAVSEGGGAQKLVF) malignant cells of patient MF311 as defined by logFC>|0.25| and adjusted p-value<0.05, as calculated by logistic regression with Bonferroni correction. **Mal clone follow-up vs patch:** Differentially expressed genes between follow-up (erythroderma) sample and patch lesion in malignant cells of patient MF311 as defined by logFC>|0.25| and adjusted p-value<0.05 using logistic regression with Bonferroni correction. **Mal clone follow-up vs plaque:** Differentially expressed genes between follow-up (erythroderma) sample and plaque lesion in malignant cells of patient MF311 as defined by logFC>|0.25| and adjusted p-value<0.05 using logistic regression with Bonferroni correction.

**Table S14. Comparison of follow-up (erythroderma) vs patch lesion in patient MF311 for each cluster, related to Figure 7.** Differential gene expression analysis within cell types of scRNA-seq analyses as defined by logFC>|0.25| and adjusted p-value<0.05, using logistic regression and Bonferroni correction.

**Table S15.** **Comparison of follow-up (erythroderma) vs plaque lesion in patient MF311 for each cluster, related to Figure 7.** Differential gene expression analysis within cell types of scRNA-seq analyses as defined by logFC>|0.25| and adjusted p-value<0.05, using logistic regression and Bonferroni correction.

**Table S16. Follow-up characterization in patient MF312, related to Figure S6.** Top 10 differentially expressed genes according to highest log fold change ordered by smallest adjusted p-value using Wilcoxon Rank Sum Test with Bonferroni correction for each cluster, as compared to the rest of the dataset in all cells for patient MF312 (integrated dataset of patch, plaque and treated lesion).

**Table S17. Comparison of treated skin vs patch lesion in patient MF312 for each cluster, related to Figure S6.** Differential gene expression analysis within cell types of scRNA-seq analyses as defined by logFC>|0.25| and adjusted p-value<0.05, using logistic regression and Bonferroni correction.

**Table S18.** **Comparison of treated skin vs plaque lesion in patient MF312 for each cluster, related to Figure S6.** Differential gene expression analysis within cell types of scRNA-seq analyses as defined by logFC>|0.25| and adjusted p-value<0.05, using logistic regression and Bonferroni correction.

**Table S19. Comparison of lesional with nonlesional MF as well as healthy control skin.** **T cell counts:** Cell counts corresponding to Figure 8. **T cell clustermarker:** Top 10 differentially expressed genes according to highest log fold change ordered by smallest adjusted p-value using Wilcoxon Rank Sum Test with Bonferroni correction for each cluster, as compared to the rest of the dataset, integrated from paired lesional and nonlesional samples of MF309 (tumor), MF311 (plaque), MF312 (plaque), as well as patients P65, P73, P84 and P90 (lesional and nonlesional skin), and 4 healthy control individuals (P112, P115, P116, P121). **Differentially expressed genes (DEGs)** between 7 paired lesional vs nonlesional MF samples in malignant cells (as defined by expression of the single top expanded TCR), regulatory T cells (TREG, *FOXP3+*), cytotoxic T Cells (Tc, *CD8A+ FOXP3-*), T helper cells (Th, *CD4+, FOXP3-*), and NK cells (TCR negative cells of cluster “NK” from Figure 8A) for each patient as defined by a logFC>|0.25| and adjusted p<0.05 using logistic regression with Bonferroni correction. **Summary** of differential gene expression analysis of 6 selected marker genes in T cell populations, shown for all 7 patients, and for each MF patient.

**Table S20. Comparison of 7 nonlesional MF with 4 healthy control samples.** Differentially expressed genes (nonlesional MF vs healthy control skin) in regulatory T cells (TREG, *FOXP3+*), cytotoxic T Cells (Tc, *CD8A+ FOXP3-*), T helper cells (Th, *CD4+, FOXP3-*), and NK cells (TCR negative cells of cluster “NK” from Figure 8A).

**Table S21. Comparison of lesional with nonlesional MF as well as healthy control skin.** **Cell counts:** Cell counts of all clustered celltypes of the integrated dataset, corresponding to Figure S8. **Clustermarker:** Top 10 differentially expressed genes according to highest log fold change ordered by smallest adjusted p-value using Wilcoxon Rank Sum Test with Bonferroni correction for each cluster, as compared to the rest of the dataset, integrated from paired lesional and nonlesional samples of MF309 (tumor), MF311 (plaque), MF312 (plaque), as well as patients P65, P73, P84 and P90 (lesional and nonlesional skin), and 4 healthy control individuals (P112, P115, P116, P121).

**Table S22. Comparison of lesional with nonlesional MF for each cluster, corresponding to Figure S8.** Differential gene expression analysis within cell types of scRNA-seq analyses as defined by logFC>|0.25| and adjusted p-value<0.05, using logistic regression and Bonferroni correction.

**Table S23. Comparison of nonlesional MF with healthy control skin for each cluster, corresponding to Figure S8.** Differential gene expression analysis within cell types of scRNA-seq analyses as defined by logFC>|0.25| and adjusted p-value<0.05, using logistic regression and Bonferroni correction.

**SUPPLEMENTARY REFERENCES**

1. Lun AT, McCarthy DJ, Marioni JC. A step-by-step workflow for low-level analysis of single-cell RNA-seq data with Bioconductor. F1000Res. 2016;5:2122.

2. Butler A, Hoffman P, Smibert P, Papalexi E, Satija R. Integrating single-cell transcriptomic data across different conditions, technologies, and species. Nat Biotechnol. 2018;36(5):411-20.

3. Stuart T, Butler A, Hoffman P, Hafemeister C, Papalexi E, Mauck WM, 3rd, et al. Comprehensive Integration of Single-Cell Data. Cell. 2019;177(7):1888-902 e21.

4. Hafemeister C, Satija R. Normalization and variance stabilization of single-cell RNA-seq data using regularized negative binomial regression. Genome Biol. 2019;20(1):296.

5. Blighe K, Rana S, Lewis M. EnhancedVolcano: Publication-ready volcano plots with enhanced colouring and labeling. 1.6.0 ed2020. https://github.com/kevinblighe/EnhancedVolcano.

6. Efremova M, Vento-Tormo M, Teichmann SA, Vento-Tormo R. CellPhoneDB: inferring cell-cell communication from combined expression of multi-subunit ligand-receptor complexes. Nat Protoc. 2020;15(4):1484-506.

7. Csardi G, Nepusz T. The igraph software package for complex network research. InterJournal, Complex Systems. 2006:1695 https://igraph.org.

8. Bruggen MC, Bauer WM, Reininger B, Clim E, Captarencu C, Steiner GE, et al. In Situ Mapping of Innate Lymphoid Cells in Human Skin: Evidence for Remarkable Differences between Normal and Inflamed Skin. J Invest Dermatol. 2016;136(12):2396-405.
